# Molecular and functional heterogeneity of IL-10-producing CD4^**+**^ T cells

**DOI:** 10.1038/s41467-018-07581-4

**Published:** 2018-12-21

**Authors:** Leonie Brockmann, Shiwa Soukou, Babett Steglich, Paulo Czarnewski, Lilan Zhao, Sandra Wende, Tanja Bedke, Can Ergen, Carolin Manthey, Theodora Agalioti, Maria Geffken, Oliver Seiz, Sara M. Parigi, Chiara Sorini, Jens Geginat, Keishi Fujio, Thomas Jacobs, Thomas Roesch, Jacob R. Izbicki, Ansgar W. Lohse, Richard A. Flavell, Christian Krebs, Jan-Ake Gustafsson, Per Antonson, Maria Grazia Roncarolo, Eduardo J. Villablanca, Nicola Gagliani, Samuel Huber

**Affiliations:** 1 I. Department of Medicine, University Medical Center Hamburg-Eppendorf, 20246 Hamburg, Germany; 2 Department of General, Visceral and Thoracic Surgery, University Medical Center Hamburg-Eppendorf, 20246 Hamburg, Germany; 3 Immunology and Allergy Unit, Department of Medicine, Solna, Karolinska Institute and University Hospital, 17176 Stockholm, Sweden; 4 Institute of Transfusion Medicine, University Medical Center Hamburg-Eppendorf, 20246 Hamburg, Germany; 5 INGM-National Institute of Molecular Genetics “Romeo ed Enrica Invernizzi”, 20122 Milan, Italy; 6 Department of Allergy and Rheumatology, Graduate School of Medicine, The University of Tokyo, 113-8655 Tokyo, Japan; 7 Department of Immunology, Bernhard-Nocht-Institute of Tropical Medicine, 20359 Hamburg, Germany; 8 Department for Interdisciplinary Endoscopy, University Medical Center Hamburg-Eppendorf, 20246 Hamburg, Germany; 9 Department of Immunobiology, School of Medicine, Yale University, New Haven, CT 06520 USA; 10 Howard Hughes Medical Institute, Yale University School of Medicine, New Haven, CT 06520 USA; 11 III. Department of Medicine, University Medical Center Hamburg-Eppendorf, 20246 Hamburg, Germany; 12 Department of Biosciences and Nutrition, Karolinska Institutet, 17177 Stockholm, Sweden; 13 Division of Stem Cell Transplantation and Regenerative Medicine, Department of Pediatrics, ISCBRM, Stanford School of Medicine, 94304 Stanford, CA USA

## Abstract

IL-10 is a prototypical anti-inflammatory cytokine, which is fundamental to the maintenance of immune homeostasis, especially in the intestine. There is an assumption that cells producing IL-10 have an immunoregulatory function. However, here we report that IL-10-producing CD4^+^ T cells are phenotypically and functionally heterogeneous. By combining single cell transcriptome and functional analyses, we identified a subpopulation of IL-10-producing Foxp3^neg^ CD4^+^ T cells that displays regulatory activity unlike other IL-10-producing CD4^+^ T cells, which are unexpectedly pro-inflammatory. The combinatorial expression of co-inhibitory receptors is sufficient to discriminate IL-10-producing CD4^+^ T cells with regulatory function from others and to identify them across different tissues and disease models in mice and humans. These regulatory IL-10-producing Foxp3^neg^ CD4^+^ T cells have a unique transcriptional program, which goes beyond the regulation of IL-10 expression. Finally, we found that patients with Inflammatory Bowel Disease demonstrate a deficiency in this specific regulatory T-cell subpopulation.

## Abstract (web summary)

Tr1 cells are considered an immunosuppressive CD4 T cell population producing IL-10. Here the authors show that IL-10 is insufficient for Tr1 immunosuppression, define surface markers and transcriptional program of the immunosuppressive subset within Tr1, and reveal its deficiency in patients with IBD.

## Introduction

Immune mediated inflammatory diseases (IMIDs) are characterized by a dysregulated immune response and non-healing tissue damage, which promotes a vicious cycle leading to chronic disease. What breaks immunological tolerance in these diseases is unknown and therefore medical therapies currently used to treat IMIDs are as of yet, not curative. Mouse studies have shown that regulatory CD4^+^ T cells and the production of the anti-inflammatory cytokine interleukin-10 (IL-10) represent fundamental mechanisms to maintain the immunological tolerance especially in the intestine. Moreover, human genetics studies have shown that polymorphisms in genes associated with the regulatory mechanisms of CD4^+^ T cells, such as interleukin-10 (*IL10*) and *IL10RA* are associated with early onset intestinal inflammation^1,2^. Thus, a defect in these mechanisms could be involved in the pathogenesis of IMIDs, especially in inflammatory bowel disease (IBD). Nevertheless, in contrast to what would be expected on the basis of these data, IBD patients do not show an obvious defect in IL-10 production^3–5^. One hypothesis to explain this discrepancy, would be that only a subpopulation of IL-10-producing CD4^+^ T cells has regulatory activity. Therefore, the quantification of all IL-10-producing CD4^+^ T cells could have been misleading, since it does not allow the quantification of this potentially hidden subpopulation. An example supporting this hypothesis is represented by one type of regulatory T cells, namely Foxp3^+^ CD4^+^ T cells. These cells have been subdivided into subpopulations based on their heterogeneous regulatory activity. This stratification was essential to understand their contribution to the prognosis of patients with colorectal cancer^6^. Despite this, no reproducible defect in number or function of Foxp3^+^ regulatory T cells could be observed in patients suffering from IBD^7–9^.

Also, IL-10-producing Foxp3^negative (neg)^ CD4^+^ T cells, usually referred to as T regulatory type 1 cells (T_R_1), have a powerful regulatory activity. However, whether this population of cells is a functional homogenous population across different tissues and species remains unknown. Different groups have described that several surface molecules, including co-inhibitory receptors such as LAG-3, PD1, and TIM-3 and also other integrins and chemokines such as CD49b and CCR5, can be expressed by IL-10-producing Foxp3^neg^ CD4^+^ T cells^10–19^. Notably, we and others already observed in distinct studies that not all IL-10-producing CD4^+^ T cells co-express LAG-3 and CD49b^11^ or TIM-3, TIGIT, PD1, and CCR5^12,20^. These data already suggested a potential functional heterogeneity, especially considering that co-inhibitory receptors are not just surface markers, but they also fulfill a regulatory function. However, it remains to be addressed whether there is a significant difference between those IL-10-producing Foxp3^neg^ CD4^+^ T cells, which express the surface markers and those which, despite IL-10 expression, do not express them.

By studying IL-10-producing Foxp3^neg^ CD4^+^ T cells, several transcriptional factors that regulate *Il10* expression have been identified. Blimp1 and C-maf^21^, through EGR-2^22,23^, promote the transactivation of the *Il10* gene. More recently, IRF1, BATF, Eomes, and T-cell receptor induced ITK were shown to be key factors in the development of these cells^24–26^. However, the transcriptional program that goes beyond the regulation of IL-10 and defines the identity of IL-10-producing Foxp3^neg^ regulatory CD4^+^ T cells has not been yet identified.

Here, by combining transcriptomic analysis at the resolution of single cells and functional experiments in mouse and humans we have shown that IL-10-producing Foxp3^neg^ CD4^+^ T cells are a functionally heterogeneous population of cells. The combinatorial expression of co-inhibitory receptors allowed the identification of a subpopulation with a regulatory function. This subpopulation displayed a unique molecular program. Finally IBD patients showed a selective paucity of these regulatory cells.

## Results

### IL-10-producing CD4^+^ T cells are heterogeneous

**Fig. 1:**
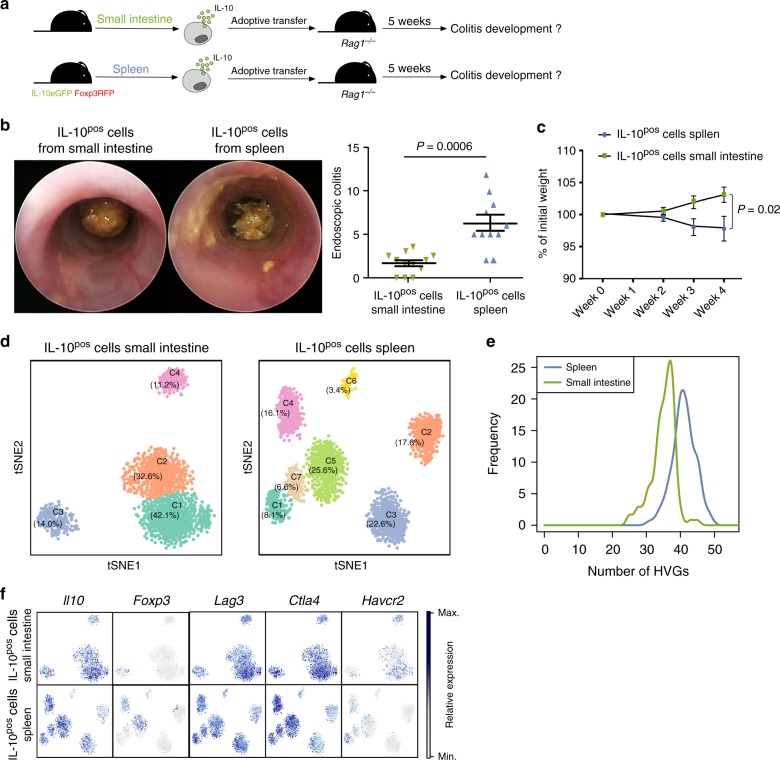
Mouse IL-10-producing CD4^+^ T cells are heterogeneous. **a**–**c** IL-10-producing Foxp3^neg^ CD4^+^ T cells (IL-10^pos^) were isolated from small intestine or spleen of aCD3-treated IL-10^eGFP^ Foxp3^mRFP^ double reporter mice. Cells were transferred into lymphopenic hosts and colitis development was assessed by weight loss (**c**) and endoscopic colitis score (**b**) 5 weeks upon transfer (IL-10^pos^ cells small intestine *n* = 12; IL-10^pos^ cells Spleen *n* = 11; lines indicate mean ±SEM). Results are cumulative of three independent experiments. A Mann–Whitney *U* test was used to calculate significance. **d** *t-*SNE analysis of single cell RNA sequencing of IL-10^pos^ cells (including Foxp3^+^ cells) isolated from small intestine or spleen of aCD3-treated IL-10^eGFP^ Foxp3^mRFP^ double reporter mice. **e** Bootstrap analysis of HVGs in single cell RNA sequencing data of IL-10^pos^ cells from small intestine and spleen. **f** Expression of indicated genes in *t-*SNE analysis

It has been assumed that all IL-10-producing T cells are a functionally homogenous population of anti-inflammatory cells. To challenge this assumption, we isolated IL-10-producing (IL-10^positive (pos)^) Foxp3^neg^ CD4^+^ T cells - we excluded Foxp3 expressing cells, as their functional heterogeneity has been already described - from small intestine and spleen using IL-10^eGFP^, Foxp3^mRFP^ double reporter mice upon in vivo expansion of this cell population via anti-CD3 antibody (mAb) treatment^11,27–29^. The function of these cells was then assessed in a T-cell mediated transfer colitis mouse model^27,28,30^. Unexpectedly, transfer of splenic IL-10-producing Foxp3^neg^ CD4^+^ T cells caused colitis upon transfer, while small intestinal IL-10-producing Foxp3^neg^ CD4^+^ T cells did not cause colitis as expected (Fig. 1a–c).

These data could be explained at least by two not mutually exclusive hypotheses: (i) only intestinal IL-10-producing Foxp3^neg^ CD4^+^ T cells have a regulatory activity or (ii) splenic IL-10-producing Foxp3^neg^ CD4^+^ T cells are a functionally heterogeneous population of cells consisting of a minority of suppressive cells that are outnumbered by a majority of inflammatory cells. To test the potential heterogeneity of IL-10-producing Foxp3^neg^ CD4^+^ T cells, we performed single cell RNA sequencing (scRNA-seq), using two complementary methods. As a control, we included IL-10-producing Foxp3^+^ CD4^+^ T cells (Supplementary Figure 1A). First, we used SMART-seq2 scRNA-seq protocol, which provides good sequencing depth, but few cells to analyze (Supplementary Figure 1). After quality control and excluding cells in G2 cell cycle or S-phase, we analyzed 138 small intestinal and 388 splenic IL-10^eGFP+^ CD4^+^ T cells. We validated that 92% of eGFP^+^ sorted cells indeed expressed *Il10*. Furthermore, there was a robust correlation between *Il10* and *eGfp* (Supplementary Figure 1B). We then performed a *t*-distributed stochastic neighbor embedding (*t*-SNE) analysis and found that the cells isolated from the spleen form more distinct clusters than those isolated from the intestine (4 clusters in spleen vs. 2 clusters in small intestine, Supplementary Figure 1C). To further strengthen this observation, we performed a bootstrap analysis by selecting 100 random cells and computing the number of highly variable genes (HVGs) in each organ. We observed that IL-10-producing cells in the spleen have more variable genes than those in the intestine (Supplementary Figure 1D). Taken together, these analyses suggest a higher heterogeneity of IL-10-producing T cells isolated from the spleen compared to the small intestine (Supplementary Figure 1).

We next used the Drop-seq based 10X technology to validate these data. We analyzed 1379 small intestinal and 1665 splenic IL-10^eGFP+^ CD4^+^ T cells after quality filtering and normalization. As observed before, the *t*-SNE analysis revealed more distinct clusters amongst IL-10^eGFP+^ CD4^+^ T cells derived from the spleen than from the small intestine (Fig. 1d). In particular, cells from the small intestine separated into 4 different clusters (SI C1-C4), among which the two largest clusters (SI C1 and C2) were relatively similar based on genes that were expressed. In the spleen, however, cells segregated into 7 clusters (Sp C1-C7). Furthermore, the number of HVGs was higher in IL-10-producing cells from the spleen compared to the small intestine (Fig. 1e). A further analysis revealed that both Sp C5 and Sp C4 contained *Foxp3* expressing cells, confirming the transcriptional heterogeneity of Foxp3^+^ T cells (Fig. 1f). Interestingly, Sp C5 was defined by high expression of *Ikzf2* (encoding HELIOS) as well as *Il2ra*, *Ctla4*, and *Icos*, which are central hallmarks of Foxp3^+^ Treg cells^31–35^, while these genes were lower in Sp C4 (Supplementary Figure 2). Finally, we observed that the expression of co-inhibitory receptors, which have been shown to be expressed on T_R_1 cells, such as *Lag3, Ctla4*, and *Havcr2* (encoding TIM-3) was different across distinct clusters (Fig. 1f).

**Fig. 2:**
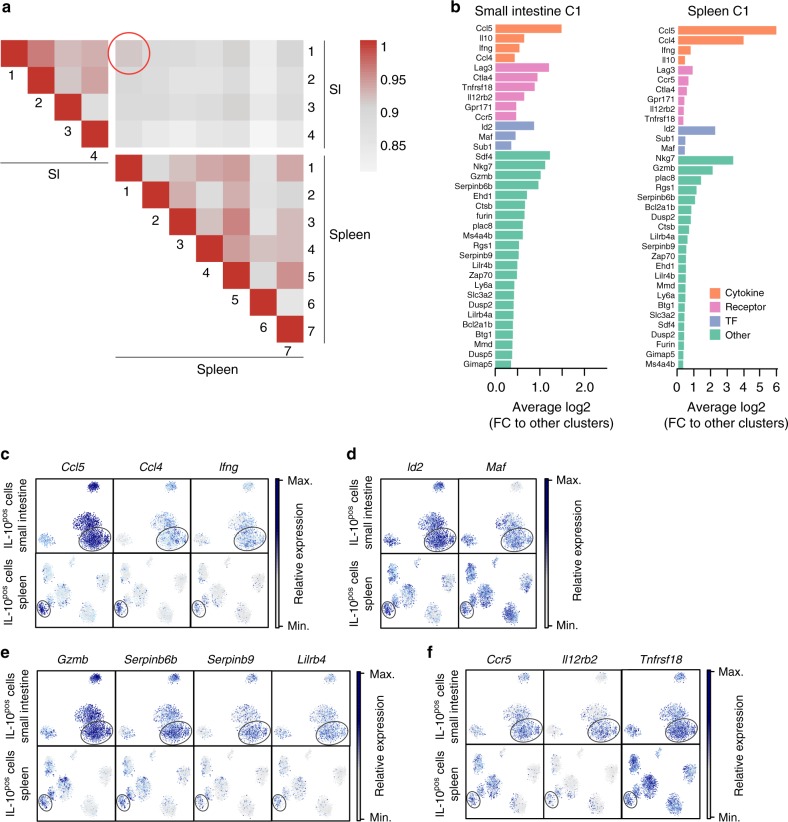
Splenic IL-10-producing CD4^+^ T cells contain a regulatory cluster. **a** Spearman correlation coefficient between each cluster from small intestine and spleen. **b** Fold change analysis of genes enriched in indicated clusters compared to other clusters that were amongst the 100 most enriched genes. **c** Expression of indicated genes encoding cytokines and chemokines. **d** Expression of indicated genes encoding transcription factors. **e** Expression of indicated genes encoding other factors. **f** Expression of indicated genes encoding surface receptors

Collectively, these data revealed a remarkable degree of heterogeneity amongst splenic IL-10-producing Foxp3^neg^ CD4^+^ T cells. Thus, it could also be possible that these subpopulations might have different functions: pro- or anti-inflammatory. From here on we focused on the identification of cell clusters with an anti-inflammatory phenotype amongst splenic IL-10-producing Foxp3^neg^ CD4^+^ T cells. To identify this cluster, we analyzed the similarity between splenic and small intestinal cell clusters: the potentially hidden regulatory cluster in the spleen might resemble the anti-inflammatory clusters present in the intestine. To this end, we calculated the Spearman correlation coefficient between each cluster from small intestine and spleen (Fig. 2a). SI C1 and Sp C1 had the highest similarity (0.912). Fold change analysis of genes highly enriched in those clusters, revealed that 35 out of 100 top enriched genes were shared by both clusters (Fig. 2b). Amongst these, we found cytokine-encoding genes, such as *Ifng* and *Il10* (Figs. 1f and 2b, c). High *Il10* expression was accompanied by high expression of *Maf*, which is a crucial transcription factor for *Il10* expression^36^. *Id2* was also very highly expressed by both clusters and has already been linked to the maintenance of regulatory T cells^37,38^ (Fig. 2b, d). Besides secretion of IL-10, granzyme secretion is another important suppressive mechanism of human and mouse T_R_1 cells^39,40^. Interestingly, *Gzmb* was indeed one gene defining both clusters (Fig. 2b, e). Concurrent with the high expression of *Gzmb*, both SI C1 and Sp C1 were defined by high expression of *Serpinb6b* and *Serpinb9* (Fig. 2b, e). *Serpinb* proteins have been demonstrated to be essential to protect granzyme B-secreting regulatory T cells from self-inflicted damage^41,42^. Also, *Lilrb4* has been linked with regulatory functions in T cells and other immune cell subsets, even though much less understood^43–45^, and was more highly expressed in Sp C1 and SI C1 compared to other clusters (Fig. 2b, e). Finally, we found that both Sp C1 and SI C1 were characterized by high expression of *Lag3*, *Ccr5*, *Ctla4, Il12rb2*, and *Tnfrsf18* (Fig. 1f, Fig. 2b, f). LAG-3, CTLA4, and CCR5 are known surface markers of T_R_1 cells^11,12^ and IL12Rβ2^46,47^ and GITR (encoded by *Tnfrsf18*) play a key role in inducing regulatory cells and their function^48–50^.

Taken together, IL-10-producing Foxp3^neg^ CD4^+^ T cells are a transcriptionally heterogeneous cell population. Furthermore, one cluster of splenic IL-10-producing Foxp3^neg^ CD4^+^ T cells resembled the main cluster seen in the small intestine, and it is characterized by expression of genes associated with T_R_1 cell biology.

### Co-inhibitory receptors identify a regulatory subpopulation

**Fig. 3:**
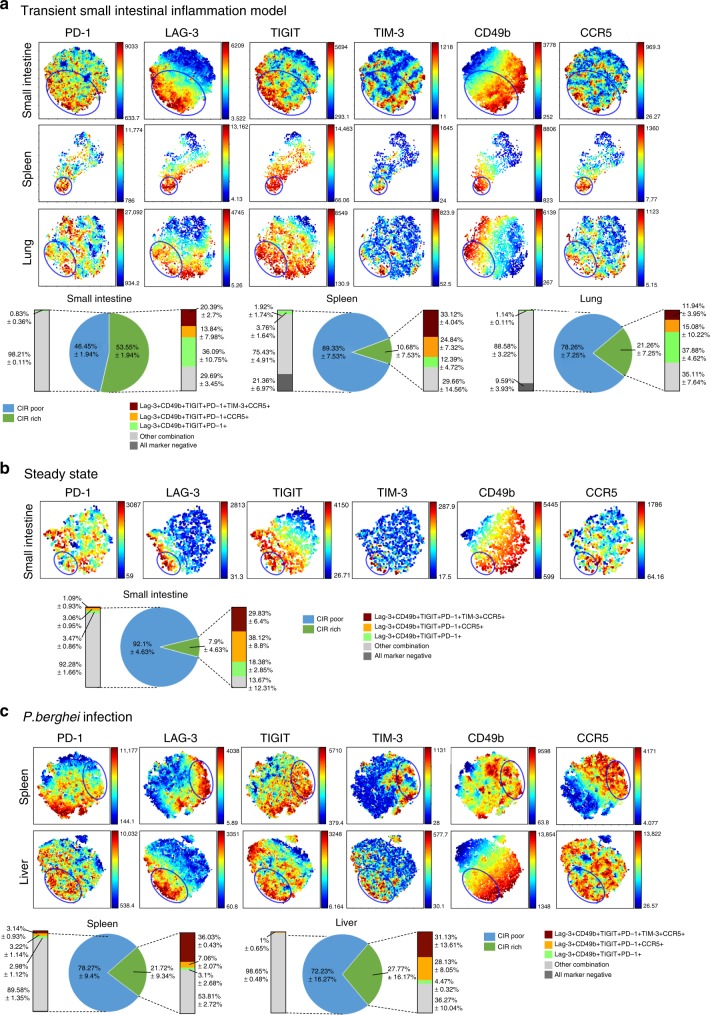
CIR identify regulatory IL-10-producing CD4^+^ T cells. *vi*SNE analysis of IL-10^pos^ Foxp3^neg^ CD4^+^ T cells. Clustering is based on MFI of PD-1, LAG-3, TIGIT, TIM-3, CD49b, and CCR5. Blue circle indicates co-inhibitory receptor rich (CIR rich) region. **a** Analysis of cells from small intestine, spleen, and lung of aCD3-treated IL-10^eGFP^ Foxp3^mRFP^ double reporter mice (*n* = 4). Data are representative of two independent experiments. **b** Analysis of untreated IL-10^eGFP^ Foxp3^mRFP^ double reporter mice (*n* = 3). Data are representative of three independent experiments. **c** Analysis of *P. berghei* infected IL-10^eGFP^ Foxp3^mRFP^ double reporter mice (*n* = 3). Data are representative of two independent experiments

We aimed to further test, if splenic IL-10-producing Foxp3^neg^ CD4^+^ T cells are a functionally heterogeneous population of cells. To this end, we had to be able to isolate the candidate suppressive population/s of cells. Notably, among the HVGs previously described, we found genes encoding for co-inhibitory receptors and other T_R_1 cell associated surface markers. For instance, *Lag3* and *Ccr5* were highly expressed in both Sp C1 and SI C1 (Fig. 2b, f). Additionally, *Havcr2* (encoding TIM-3) was highly expressed by cells in Sp C1 (Fig. 1f; Supplementary Figure 2). Thus, we analyzed whether the simultaneous expression of several surface molecules allows the isolation of cells enriched in the Sp C1 population. First we confirmed that co-inhibitory receptors (i.e. LAG-3, TIGIT, TIM-3, and PD-1) and T_R_1 associated surface markers (i.e. CD49b and CCR5) were most abundantly expressed by IL-10-producing Foxp3^neg^ CD4^+^ T cells compared to IL-10 negative T cells. Second, we found in all analyzed organs and models that some of the IL-10-producing Foxp3^neg^ CD4^+^ T cells were negative for co-inhibitory markers (Supplementary Figure 3). Then, in order to visualize the complexity of this multi-parameter analysis we used *vi*SNE analysis^51^. We found that the expression of the analyzed markers varied drastically across IL-10-producing Foxp3^neg^ CD4^+^ T cells within one and different tissues (Fig. 3a, blue circle). However, we consistently identified a subpopulation that contained cells enriched for all co-inhibitory receptors and the other surface molecules tested. From here on we refer to this IL-10-producing Foxp3^neg^ CD4^+^ T cells as co-inhibitory receptor (CIR) rich population. Interestingly, one major difference between the analyzed tissues was the frequency of CIR rich CD4^+^ T cells. While in the small intestine of anti-CD3 treated animals more than half of the analyzed cells were CIR rich, these cells represented a minority in the spleen, where only 10% of them were found (Fig. 3a, right panel). Furthermore, we confirmed our findings under steady state conditions in small intestine and during *P. berghei* infection, a model of Malaria^52^, in spleen and liver (Fig. 3b, c).

Thus, using a multi-parameter analysis we identified a subpopulation of IL-10-producing Foxp3^neg^ CD4^+^ T cells, which express simultaneously several IL-10 associated markers across different tissues and mouse models.

### CIR rich cells are suppressive

Next, we tested whether IL-10-producing Foxp3^neg^ CIR rich CD4^+^ T cells have a distinct function from IL-10-producing Foxp3^neg^ CIR poor cells. To this end, we had to be able to isolate these CIR rich and CIR poor cells from spleens of anti-CD3 treated or *P. berghei* infected IL-10^eGFP^ Foxp3^mRFP^ double reporter mice and then tested their suppressive capacity. Since *vi*SNE cannot be used for FACS sorting, we assessed the specificity and sensitivity of different sets of markers to isolate CIR rich cells. The result of this analysis showed that among several combinations of markers, the co-expression of IL-10^eGFP^, CD49b, and LAG-3 had the highest sensitivity and still a specificity of over 90% for detecting CIR rich cells. We also tested whether high IL-10 expression is sufficient to identify IL-10-producing Foxp3^neg^ CIR rich CD4^+^ T cells. However, this had the lowest sensitivity and specificity among all the combinations of markers tested (Supplementary Figure 4A and B).

**Fig. 4:**
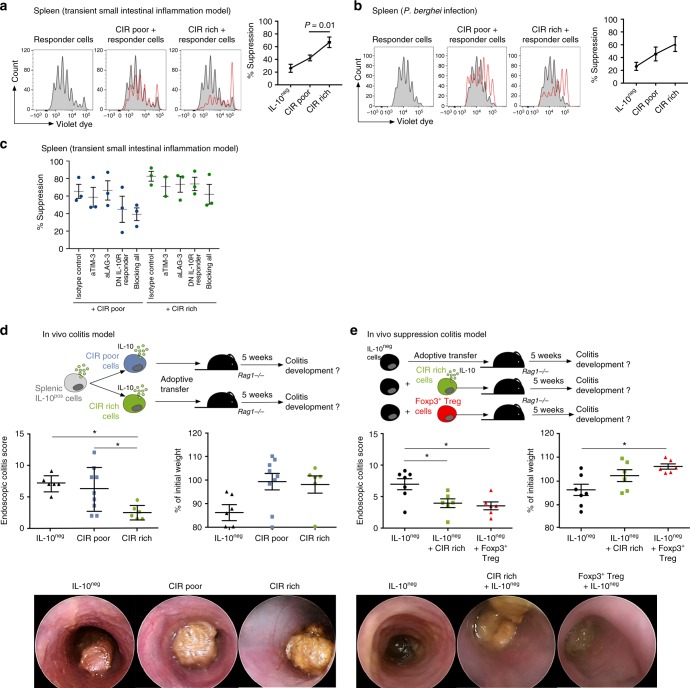
CIR rich CD4^+^ T cells have a high suppressive capacity. **a** In vitro suppression of co-inhibitory receptor rich (CIR rich) and co-inhibitory receptor poor (CIR poor) IL-10^pos^ Foxp3^neg^ CD4^+^ T cells isolated from spleen of aCD3-treated IL-10^eGFP^ Foxp3^mRFP^ double reporter mice. Representative histograms of five independent experiments, a paired *T-*test was used to calculate significance. **b** In vitro suppression of CIR rich and CIR poor IL-10^pos^ Foxp3^neg^ CD4^+^ T cells isolated from spleen of *P. berghei* infected IL-10^eGFP^ Foxp3^mRFP^ double reporter mice. Representative histograms of four independent experiments. **c** In vitro suppression of CIR rich and CIR poor IL-10^pos^ Foxp3^neg^ CD4^+^ T cells isolated from spleen of aCD3-treated IL-10^eGFP^ Foxp3^mRFP^ reporter mice. TIM-3 and LAG-3 were blocked using blocking antibodies. To block IL-10 receptor signaling responder T cells were isolated from IL-10R dominant negative mice (DN IL-10R Responder). Results are cumulative of three independent experiments. **d** CIR rich and CIR poor IL-10^pos^ Foxp3^neg^ CD4^+^ T cells and IL-10^neg^ CD4^+^ T cells were isolated from spleen of aCD3-treated IL-10^eGFP^ Foxp3^mRFP^ double reporter mice. Cells were transferred into lymphopenic hosts and colitis development was assessed by weight loss and endoscopic colitis score 5 weeks upon transfer (IL-10^neg^ *n* = 6; CIR poor *n* = 9; CIR rich *n* = 6; lines indicate mean±SEM). Results are cumulative of three independent experiments. One-way ANOVA (post-test Tukey) was used to calculate significance (*p < 0.05). **e** CIR rich IL-10-producing Foxp3^neg^ CD4^+^ T cells, IL-10^neg^ CD4^+^ T cells and Foxp3^+^ Treg cells were isolated from spleen of aCD3-treated IL-10^eGFP^ Foxp3^mRFP^ double reporter mice. IL-10^pos^ CIR rich or Foxp3^+^ Treg cells were co-transferred with IL-10^neg^ CD4^+^ T cells and colitis development was assessed by weight loss and endoscopic colitis score 5 weeks upon transfer (IL-10^neg^ *n* = 7; IL-10^neg^ + CIR rich *n* = 6; IL-10^neg^ + Foxp3^+^ Treg *n* = 6; lines indicate mean±SEM). Results are cumulative of three independent experiments. One-way ANOVA (post-test Tukey) was used to calculate significance (*p < 0.05)

After having established the use of CD49b/LAG-3/IL-10 as the most efficient gating strategy, we then FACS-sorted splenic CIR rich and CIR poor cells (Supplementary Figure 5) from both anti-CD3 treated and *P. berghei* infected mice and tested their suppressive capacity in vitro. We found that CIR rich cells had a high in vitro suppressive capacity in contrast to CIR poor cells (Fig. 4a, b). There was a correlation, by trend and in some organs, between IL-10 expression and CIR rich cells (Supplementary Figure 4C). Therefore, in order to assess whether differences in IL-10 production might account for the different suppressive activity we did two additional experiments. We performed an in vitro suppressive experiment using CIR rich and CIR poor cells isolated from liver of *P. berghei* infected mice: In this organ the IL-10^eGFP^ MFI among CIR rich and CIR poor is equal (Supplementary Figure 4C). Also in this case, CIR rich cells had a superior in vitro suppressive capacity compared to IL-10-producing Foxp3^neg^ CIR poor cells (Supplementary Figure 4D). Furthermore, we performed in vitro suppressive assays comparing IL-10^intermediate^ CIR rich with IL-10^intermediate^ CIR poor cells isolated form the spleen of the anti-CD3 treated mice in order to assure that we analyze populations with equal IL-10 production. We found that also the IL-10^intermediate^ CIR rich had a higher suppressive capacity compared to CIR-poor cells (Supplementary Figure 4E).

We next wondered whether CIR rich and CIR poor cells differed only in their suppressive activity or also in the mechanisms of function. Due to the co-expression of several co-inhibitory receptors, we hypothesized that CIR rich cells have a large portfolio of regulatory mechanisms (e.g. co-inhibitors receptor). Indeed, we found that blockage of either LAG-3, or TIM-3 or IL-10 receptor signaling alone only slightly affected the suppressive capacity of CIR rich cells. However, blocking all three, LAG-3, TIM-3, and IL-10 receptor signaling, reduced the suppressive capacity of CIR rich cells to the level of CIR-poor cells (Fig. 4c).

We then divided the splenic IL-10-producing Foxp3^neg^ CD4^+^ T cells, which promoted colitis when transferred as one population in *Rag1* deficient mice (Fig. 1a–c), into CIR rich and CIR poor cells and tested their pathogenicity in vivo. CIR rich cells were indeed non-pathogenic, while CIR poor cells induced moderate to severe colitis (Fig. 4d). As control, we used IL-10^neg^ CD4^+^ T cells, which induced severe colitis as expected. To finally test the suppressive capacity of CIR rich cells, we co-transferred these cells together with IL-10^neg^ CD4^+^ T cells into lymphopenic hosts (Fig. 4e). CIR rich cells were able to prevent intestinal disease to a similar extent as Foxp3^+^ Treg cells (Fig. 4e). Additionally, we tested if the anti-colitogenic effect of CIR cells was dependent on IL-10. To this end, we co-transferred CIR cells with IL-10^neg^ CD4^+^ T cells isolated from either wild type or transgenic mice with T-cell specific over expression of a dominant negative IL-10Rα (IL-10R^impaired^), in which IL-10 signaling is largely impaired^29^. In this case, CIR rich failed to supress disease mediated by effector T cells with impaired IL-10 signaling (Supplementary Figure 6A). These data indicate that the suppressive capacity of CIR cells during colitis depends on IL-10.

Finally, to investigate why CIR poor cells were pathogenic (Fig. 4d), we tested their stability. We found that CIR poor cells lose the expression of IL-10 upon in vitro re-stimulation, while CIR rich cells retained the expression of IL-10 (Supplementary Figure 6B). Thus CIR poor cells have a less stable phenotype compared to CIR rich cells.

In summary, we demonstrated that only a subpopulation of splenic IL-10-producing Foxp3^neg^ CD4^+^ T cells has anti-inflammatory function. This subpopulation is characterized by the co-expression of co-inhibitory receptors and is present across different organs and disease models. It relies on several mechanisms of suppression, but IL-10 is fundamental during colitis. Thus, these data support the hypothesis that IL-10-producing Foxp3^neg^ CD4^+^ T cells are a functionally heterogeneous population of cells.

### Transcriptional program of CIR rich CD4^+^ T cells

**Fig. 5:**
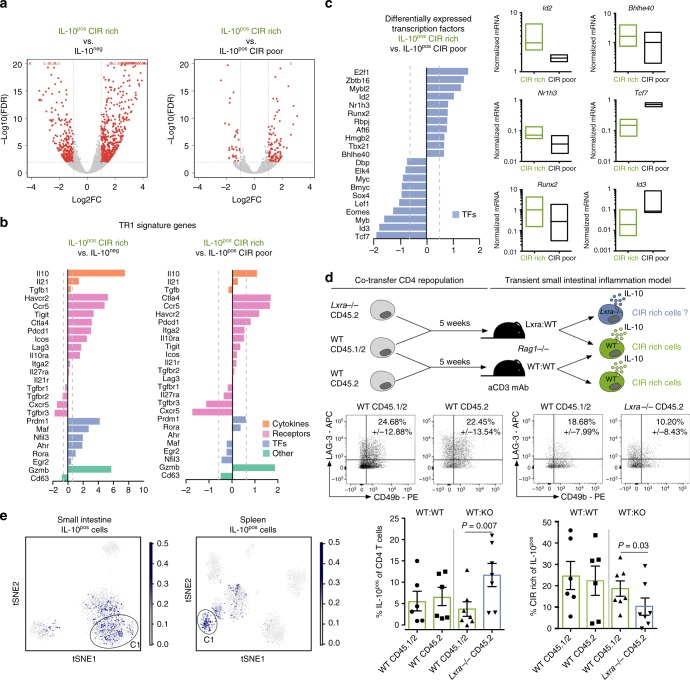
CIR rich CD4^+^ T cells have a distinct transcriptional program. **a** Volcano plots of bulk RNA sequencing data of indicated populations (IL-10^pos^ CIR rich *n* = 2; IL-10^pos^ CIR poor *n* = 2; IL-10^neg^ *n* = 2). **b** Expression of known T_R_1 signature genes comparing IL-10^pos^ CIR rich with IL-10^neg^ and IL-10^pos^ CIR rich with IL-10^pos^ CIR poor. **c** Differentially expressed transcription factors between IL-10^pos^ CIR rich and IL-10^pos^ CIR poor. mRNA expression of indicated genes normalized to *Hprt* from at least three independent experiments (separated low-high, line represents median). **d** CD4^+^ T cells isolated from *Lxrα*-/- CD45.2 and wildtype CD45.2 mice were co-transferred with wildtype CD4^+^ T cells (CD45.1/2) into lymphopenic hosts. Animals (WT:KO *n* = 7; WT:WT *n* = 6) were treated with aCD3 antibodies 5 weeks upon transfer and cells were isolated from small intestine. Results are cumulative of two independent experiments. A Wilcoxon test was used to calculate significance. **e** Correlation between genes significantly higher expressed in IL-10^pos^ CIR rich versus IL-10^pos^ CIR poor cells and expression pattern of scRNA-seq data

Our data suggest that non-regulatory CD4^+^ T cells also express IL-10. Thus, the transcription factors regulating *Il10* expression might be necessary but not sufficient to define regulatory IL-10-producing Foxp3^neg^ CD4^+^ T cells. Therefore, to go beyond the transcriptional factors regulating the expression of *Il10*, we profiled the transcriptome of regulatory CIR rich cells. As control, we used CIR poor cells and IL-10 negative Foxp3^neg^ CD4^+^ T cells isolated from the spleens of the same animals. CIR cells were FACS-sorted as LAG-3, CD49b, and IL-10^eGFP High^ triple positive, while IL-10-producing Foxp3^neg^ CIR poor CD4^+^ T cells were IL-10^eGFP High^ cells not co-expressing LAG-3 and CD49b as shown in Supplementary Figure 5. As expected, we found a high number of genes (1051) differentially expressed between IL-10-producing CIR rich CD4^+^ T cells and IL-10^neg^ cells (Fig. 5a). The expression of *Il10* and most T_R_1 related surface receptors and markers was highly increased in CIR rich cells compared to IL-10^neg^ cells (Fig. 5b). Also, genes encoding transcription factors previously linked with the development of T_R_1 cells such as *Prdm1*, *Maf*, *Nfil3*, *Ahr*, and *Rora* were significantly more expressed in CIR rich cells compared to IL-10^neg^ cells (Fig. 5b). In contrast, between CIR rich and CIR poor cells only 174 genes were differentially expressed (Fig. 5a). The expression of IL-10 related transcription factors was unaltered between CIR rich and CIR poor cells, even though the expression of *Il10* was slightly higher in CIR rich cells (Fig. 5b). We therefore approached this through an unsupervised analysis. We found 11 genes encoding transcription factors with expression that was higher and 10 where it was lower in CIR rich compared to CIR poor cells. We confirmed the expression pattern of some of these factors by qPCR (Fig. 5c). Amongst transcription factors that were more highly expressed, several have been linked to cell cycle and proliferation such as *E2f1*, *Zbtb16*, and *Mybl2*, indicating a more proliferative state of CIR rich compared to CIR poor cells^53–55^. This data might also suggest that CIR rich cells have a different activation status than CIR poor cells. We therefore analyzed the expression of common activation markers of CIR rich and CIR poor in the scRNA-seq data set. However, we could not detect a distinct expression pattern of activation markers. Furthermore, we showed that CIR rich and CIR poor cells have similar CD62L and CD44 protein expression (Supplementary Figure 7). Thus, our data disprove a significant difference in the activation status between CIR poor and rich cells, but rather suggest a different proliferative status.

In addition, *Id2*, a factor that we found to be highly expressed in both Sp C1 and SI C1 in the single cell sequencing data set (Fig. 2b, d), was also amongst the transcription factors highly expressed in CIR rich cells (Fig. 5c). Another intriguing transcription factor selectively expressed in CIR rich cells was *Nr1h3* (Fig. 5d), encoding for Liver X receptor α (LXRα). LXRα has already been described in macrophages as inhibiting pro-inflammatory pathways; furthermore, new evidence shows that LXR activation can induce Foxp3^+^ regulatory T cells with superior suppressive function^56–59^. Consequently, to prove that our sequencing analysis identified relevant transcription factors for the biology of IL-10-producing Foxp3^neg^ CIR rich CD4^+^ T cells, we tested the function of LXRα. To this end, we co-transferred CD4^+^ T cells from *Lxrα*-/- mice together with congenic wild-type CD4^+^ T cells into lymphopenic hosts (gating strategy displayed in Supplementary Figure 8). This co-transfer approach helped to discriminate between cell intrinsic and extrinsic effects. After re-population of the recipients with CD4^+^ T cells we induced IL-10-producing T cells by treating the animals with anti-CD3 antibodies. Of note, the overall IL-10 production by *Lxrα*-/- CD4^+^ T cells was not reduced but it even increased. However, we found significantly less IL-10-producing Foxp3^neg^ CIR rich CD4^+^ T cells, identified for simplicity as CD49b and LAG-3 co-expressing cells, among the *Lxrα*-/- CD4^+^ T cells compared to wild type CD4^+^ T cells (Fig. 5d). These data indicate that LXRα indeed does not inhibit the development of IL-10-producing Foxp3^neg^ CD4^+^ T cells, but may affect the biology of IL-10-producing Foxp3^neg^ CIR rich CD4^+^ T cells within those (Fig. 5d).

Finally, we wanted to test which subpopulations of IL-10-producing Foxp3^neg^ CD4^+^ T cells identified by single-cell sequencing express the gene signature of CIR rich T cells identified by bulk RNA sequencing. To this end, we correlated the expression pattern of each cell from the scRNA-seq analysis with the expression pattern of CIR rich cells (Fig. 5e). We observed that cells in Sp C1 and SI C1/C2 showed higher correlation to the gene signature of CIR rich cells (Fig. 5e). This result further corroborates our previous findings that these clusters are enriched for CIR rich cells (Fig. 1f). Moreover, this analysis enabled us to link the CIR rich cells, identified by the expression of surface molecules, with clusters of cells that we identified using scRNA-seq analysis.

In summary, we identified the transcriptional program of regulatory CIR rich cells which goes beyond the regulation of IL-10.

### Deficiency of CIR rich CD4^+^ T cells in IBD

**Fig. 6:**
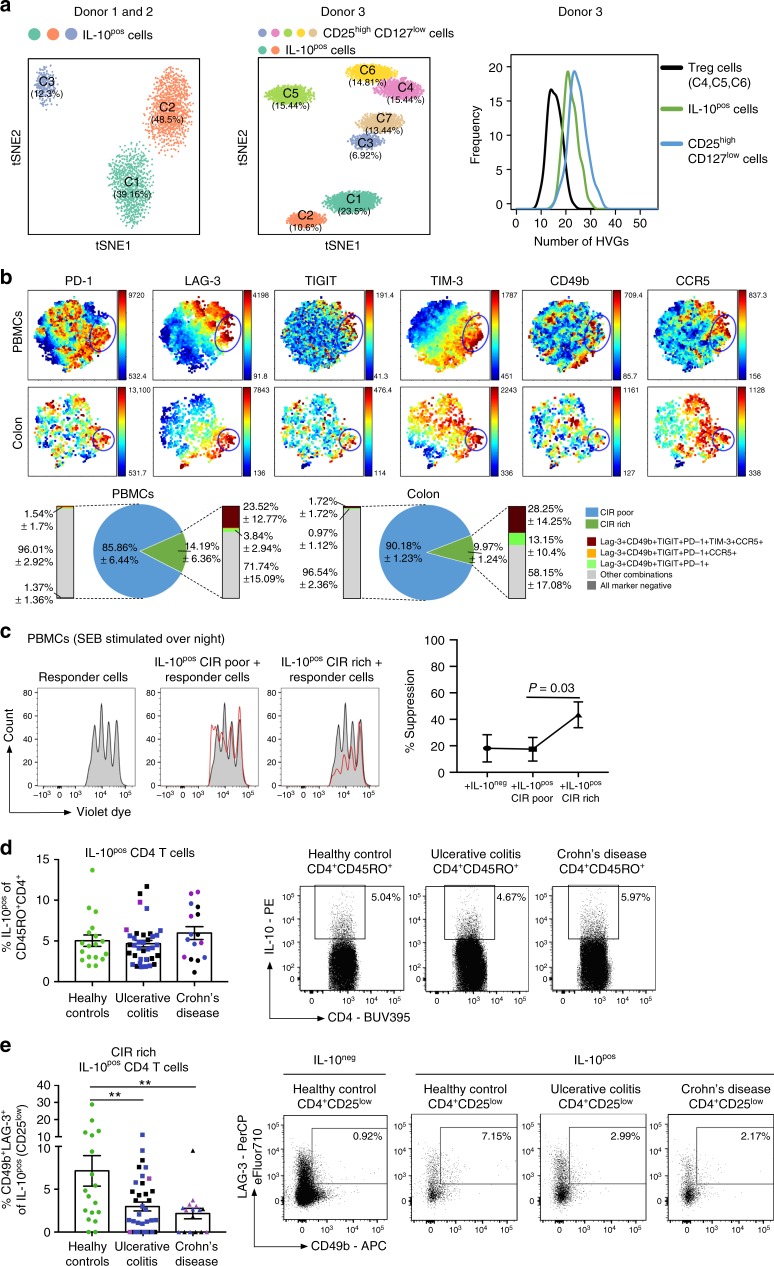
Human IL-10-producing CD4^+^ T cells are heterogeneous. **a** RNA single cell sequencing data of IL-10^pos^ cells (including Foxp3^+^ T cells) isolated from PBMCs of healthy donors (*n* = 3), stimulated with SEB overnight. **b** *vi*SNE analysis of IL-10^pos^ CD25^low^ CD4^+^ T cells from PBMCs of healthy donors (*n* = 8) and healthy colon biopsies (*n* = 4), stimulated with SEB overnight. Clustering is based on MFI of PD-1, LAG-3, TIGIT, TIM-3, CD49b, and CCR5. Blue circle indicates co-inhibitory receptor rich (CIR rich) region. **c** In vitro suppression of CIR rich and CIR poor IL-10^pos^ CD25^low^ CD4^+^ T cells isolated from PBMCs of healthy donors, stimulated with SEB overnight. Representative histograms of five independent experiments, a paired *T-*test was used to calculate significance. **d**, **e** Analysis of IL-10^pos^ CD25^low^ CD4^+^ T cells (**d**) and expression of CD49b/LAG-3 within those (**e**) of colon biopsies from IBD patients: UC active *n* = 22 (inflamed *n* = 20, blue dots; non-inflamed *n* = 17, black dots); UC Remission *n* = 2, violet dots; CD active *n* = 9 (inflamed *n* = 5, blue dots; non-inflamed *n* = 6, black dots); CD remission *n* = 5, violet dots and healthy controls (*n* = 18). Cells were stimulated with SEB overnight, One-way ANOVA (multiple comparisons) was used to calculate significance (**p < 0.005)

We next aimed to translate these findings into human biology. To that end we performed scRNA-seq analysis of IL-10-producing CD4^+^ T cells isolated from the blood (PBMCs) of three healthy donors. After quality control, cell cycle filtering and normalization, we analyzed 4438 IL-10-producing T cells pooled from donor 1 and donor 2. Moreover, we tested 5171 cells form a 3^rd^ donor and in this case we also included CD25^high^ CD127^low^ CD4^+^ T cells as control. This is a cell population, which is indeed known to be functionally heterogeneous^6^. Using *t*-SNE analysis, we identified 3 and 2 different clusters among IL-10-producing CD4^+^ T cells from donor 1/2 and donor 3, respectively (Fig. 6a). Moreover, using the analysis of donor 3, we observed that IL-10-producing CD4^+^ T cells are very distinct from CD25^high^ CD127^low^ CD4^+^ T cells. As expected^6^ these cells formed even more clusters than IL-10-producing CD4^+^ T cells (Fig. 6a and Supplementary Figure 9A). The formation of at least two clusters suggested heterogeneity of human IL-10-producing CD4^+^ T cells, albeit at a lower grade compared to the functionally heterogeneous CD25^high^ CD127^low^ CD4^+^ T cells. To further assess the heterogeneity of IL-10-producing CD4^+^ T cells, we performed bootstrap analysis of highly variable genes (HVGs). We found that IL-10-producing CD4^+^ T cells appear to be slightly less heterogeneous than CD25^high^ CD127^low^ CD4^+^ T cells. However, when we compared IL-10-producing CD4^+^ T cells with the clusters C4, C5, and C6, which are rich in *FOXP3*^high^ *IL2R*^high^ *CD127*^low^ cells and have previously been proved to be the bona-fide FOXP3^+^ Treg cells, we observed instead that IL-10-producing CD4^+^ T cells are more heterogeneous (Fig. 6a, Supplementary Figure 9B and C).

Overall, these data indicate that also human IL-10-producing CD25^low^ CD4^+^ T cells are a heterogonous population.

To further test this we then performed *vi*SNE analysis on the basis of the expression of PD-1, LAG-3, TIGIT, TIM-3, CD49b, and CCR5 in IL-10-producing CD25^low^ CD4^+^ T cells. First we confirmed that the IL-10 producing CD4^+^ T cells express significantly higher level of the tested surface molecules compared to the IL-10^neg^ CD4^+^ T cells (Supplementary Figure 10B). Then we found that a fraction of intestinal and circulatory humans CD4^+^ T cells co-expressed all the tested surface molecules, i.e. CIR cells (Fig. 6b). Furthermore, we confirmed that co-expression of only IL-10, CD49b, and LAG-3 showed the highest sensitivity to enrich for these human CIR rich cells (Supplementary Figure 10A). Additionally, we excluded the possibility that FOXP3^+^ Treg ‘contaminated’ the analyzed population of CD25^low^ memory CD4^+^ T cells (Supplementary Figure 10B). Most importantly, we observed a stronger suppressive capacity of IL-10-producing CD25^low^ CIR cells compared to CIR poor cells (Fig. 6c). Of note, there was no significant difference in IL-10 expression between IL-10-producing CD25^low^ CIR rich and CIR poor cells (Supplementary Figure 10C). Finally CIR rich cells also express a higher level of LXRα compared to CIR poor cells (Supplementary Figure 10D).

All together these findings strengthen the link between the mouse and human data and the notion that CIR cells are preserved between species.

Finally, we analyzed IL-10-producing CD25^low^ CD4^+^ T cells in IBD patients with CD and Ulcerative Colitis (UC) (Patient information: Supplementary Figure 11). We could not find paucity in total IL-10 production in CD4^+^ T cells in the colon of IBD patients, or in CD or in UC patients, compared to healthy controls (Fig. 6d). However, we found a significantly reduced frequency of IL-10-producing CD25^low^ CIR rich CD4^+^ T cells within the IL-10-producing CD25^low^ CD4^+^ T cells in IBD patients (Fig. 6e). Thus, our data indicate that IBD patients do not have a general defect in IL-10-producing CD4^+^ T cells, but a selective paucity in CIR rich cells. This defect seems to be a common feature of patients with both UC and CD (Fig. 6e).

## Discussion

We show here that IL-10-producing Foxp3^neg^ CD4^+^ T cells are a functional heterogeneous population of cells: while some of these cells have an anti-inflammatory function other are potentially pathogenic. Using the combinatorial expression of co-inhibitory receptors, it is possible to identify the anti-inflammatory IL-10-producing Foxp3^neg^ CD4^+^ T cell population. This population is conserved across different tissues and species. Finally, we revealed that the anti-inflammatory IL-10-producing Foxp3^neg^ CD4^+^ T cell population is reduced in IBD patients showing that these patients have a defect in immunological tolerance.

Several co-inhibitory receptors were reported by others and us to be expressed on IL-10-producing Foxp3^neg^ CD4^+^ T cells^11–19,22,60^. However, it was not clear whether these markers identified the same or different populations of cells. The visualization of all these co-inhibitory receptors simultaneously showed that there are IL-10-producing cells, which are rich in all the co-inhibitory receptors, tested. The size of this population varied across different tissues and species, but we were always able to find these cells. Furthermore, these cells - i.e. CIR rich cells - have a strong regulatory activity. Therefore, this population fits the original definition of T_R_1 cells, which is mainly based on the expression of IL-10 and on the functional activity^10^. It is still possible that different combinations of the same co-inhibitory receptors or the discovery of new co-inhibitory receptors will help to identify other regulatory populations. However, we think that this should not lead to the amplification of T cell nomenclature, but rather to focus on the different biological aspects of the potential subpopulations of T_R_1 cells.

We and others reported before, and confirmed as part of this study, that CD49b/LAG-3 (or PD1/CCR5) can be used to identify IL-10-producing cells within CD4^+^ T cells^11,12,16,17,61–64^. Here we extended this finding by showing that CD49b/LAG-3 can also be used to identify the regulatory subset within the functional heterogeneous population of IL-10-producing CD4^+^ cells. The specificity to enrich regulatory IL-10-producing CD4^+^ T cells is increased when more markers are combined, at the price of lower sensitivity. Thus the enrichment strategy can be adjusted according to the required purity. Interestingly, our data also show that IL-10-producing Foxp3^neg^ CIR poor are pathogenic, although they produce similar amounts of IL-10 compared to CIR rich cells. Thus, other combinations of co-inhibitory receptors could also help to further study these potentially pathogenic cells.

More importantly, we started revealing the transcriptional network going beyond the transcriptional regulation of *Il10*. By comparing IL-10-producing CD4^+^ T cells with and without regulatory activity, we diverged the network regulating only IL-10 from the one regulating the anti-inflammatory identity of these cells. By doing so, we identified several transcriptional factors, which now need a further analysis to understand their function in T_R_1 biology. An obvious candidate is LXR. It has been shown that *LXRA* and *LXRB* expression is reduced in colon of IBD patients compared to healthy controls^65^. Therefore, manipulation of LXR function and successive induction of highly suppressive IL-10-producing Foxp3^neg^ CD4^+^ T cells could be an intriguing therapeutic strategy since LXR activity can be regulated by synthetic ligands^66,67^. Of note, a recently published study showed that IL-10 expression is negatively regulated by 25^−^OHC via LXRα/β^68^. In line with these data, we also observed an increased frequency of IL-10-producing cells in *Lxra*-deficient CD4^+^ T cells compared to wild type. However the fraction of CIR rich cells was reduced, suggesting that LXR plays a role in the biology of CIR rich cells. It is possible that LXR has a different impact on the differentiation and stability of CIR rich cells, and this requires further experiments. Furthermore, our data suggest a role of Bhlhe40, which was recently shown to regulate IL-10 expression^69^. However the role of Bhlhe40 in the biology of the CIR rich CD4^+^ T cells remains to be tested.

The strong anti-inflammatory activity of T_R_1 cells, has been proved by others and by us in several preclinical mouse models of IMIDs^11,27,29,60^. Additionally, two clinical trials have already been successfully completed with the use of in vitro induced T_R_1 cells^13,70^. These successful trials, even if preliminary, exemplify the difference between a therapy with IL-10, which only partially succeeded (reviewed in^71^), and a therapy with the use of a cell which can produce the right dose of IL-10 in the target organs and more importantly, can exert regulatory activity through multiple mechanisms. Here we found, for the first time, that CIR rich cells are reduced in both UC and CD patients. These data finally justify the use a T_R_1-cell based therapy in IBD patients.

## Methods

### Mice

C57BL/6 CD45.2, C57BL/6 *Rag1*-/- CD45.1 were obtained from The Jackson Laboratory. Foxp3^mRFP^, IL-10^eGFP^ reporter mice and *Lxrα*-/- mice are described elsewhere^29,72^. Age and sex- matched littermates between 8 and 16 weeks of age were used.

### Lymphocyte isolation from the intestine of mice

Intraepithelial lymphocytes were collected after removing Peyer’s patches by incubation of the small intestine with 5 mM EDTA solution at 37 °C for 30 min. Lamina propria lymphocytes were isolated by digesting the tissue with collagenase IV (100 U; Sigma-Aldrich) at 37 °C for 45 min. The cells were further separated by a Percoll gradient (GE Healthcare).

### Lymphocyte isolation from the lung and liver of mice

Lymphocytes from lung and liver of mice were isolated by digesting the tissue with collagenase IV (100 U; Sigma-Aldrich) for 45 min at 37 °C. The cells were further separated by a Percoll gradient (GE Healthcare).

### Flow cytometry

Briefly, after immune cell isolation (described above), cells were either analyzed freshly (in case of reporter mice) or re-stimulated with PMA/Ionomycin for 3 h (non-reporter mice) or in vitro re-stimulated with SEB (1 µg/ml) to detect IL-10 cytokine secretion (in case of human samples).

Panels for FACS analysis mouse: TCRbeta (BV421, BioLegend, clone H57-597 Dilution 1:200, lot B209221), CD4 (BUV737, BD Bioscience, clone GK1.5, Dilution 1:800, lot 5100736/PE/Cy7, BioLegend, clone RM4-5, Dilution 1:400, lot B19204), CD49b (PE, BioLegend, clone Hma2, Dilution 1:100, lot B148368; FITC, BioLegend, clone Hma2, Dilution 1:100, lot B178538), LAG-3 (APC, BioLegend, clone C9B7W, Dilution 1:100, lot B163789), TIM-3 (Biotin, eBioscience, clone 8B.2C12, Dilution 1:200, lot E02985-1632), Strepdavidin (BUV395, BD Bioscience, clone NA, Dilution 1:200), TIGIT (PerCPCy5.5, eBioscience, clone GIGD7, Dilution 1:200, lot E15928-104), PD-1 (BV605, BioLegend, clone 29 F.1A12, Dilution 1:300, lot B227578), CCR5 (PECy7, BioLegend, clone HM-CCR5, Dilution 1:200, lot B220574), CD45.1 (Pacific Blue, Biolegend, clone A20, Dilution 1:400, lot B191340), CD45.2 (PECy7, BioLegend, clone 104, Dilution 1:400, lot B185406), and IL-10 (PE-TexasRed, BioLegend, clone JES5-16E3, Dilution 1:100, lot B243511). For intracellular IL-10 staining, cells were fixed with 4% PFA for 20 min at RT and permeabilized with 0.1 % NP40 solution for 4 min. Cells were stained for IL-10 for 1 h at room temperature. Staining for CD49b and LAG-3 was performed for 30 min at 37 °C.

Panels for FACS analysis human: CD45RO (BV510, BioLegend, clone UCHL1, Dilution 1:400, lot B226190), CD4 (BUV395, BD Bioscience, clone SK3, Dilution 1:200, lot 5309778), CD127 (PeCy7, BioLegend, clone A019D5, Dilution 1:400, lot B223477), CD25 (BV650, BioLegend, clone BC96, Dilution 1:100, lot B198924), CD49b (APC, eBioscience, clone P1H5, Dilution 1:50, lot 4301298), LAG-3 (PerCP eFluor710, eBioscience, clone 3DS223H, Dilution 1:20, lot 4321735), TIM-3 (BV785, BioLegend, clone F38-2E2, Dilution 1:100, lot B236242), TIGIT (AlexaFluor700, R&D Systems, Dilution 1:100, lot AEEA0215071), PD-1 (BV421, BioLegend, clone EH12.2H7, Dilution 1:100, lot B217577), CCR5 (FITC, BD Bioscience, Dilution 1:20, lot 5357814), IL-10 (PE, Miltenyi, IL-10 Secretion Assay – Detection Kit, Cat.# 130-090-434). IL-10 staining was performed using IL-10 secretion assay – detection kit in accordance with the manual (Miltenyi). Staining for CD49b and LAG-3 was performed for 30 min at 37 °C.

Samples were acquired on an LSR II flow cytometer (BD Bioscience) and analyzed using Flowjo. Additionally, data were analyzed using the Cytobank platform (*vi*SNE analysis).

### Transient small intestinal inflammation mouse model

Mice were injected with anti-CD3 (clone 2C11, 15 µg) i.p. twice every other day (injection on day 0 and day 2). Animals were killed 4 h after the second injection. Controls were injected with isotype-matched antibodies or PBS.

After CD4^+^ T cell re-population of lymphopenic hosts, animals were injected with anti-CD3 (clone 2C11) i.p. twice every other day: first injection 3 µg anti-CD3, second injection 15 µg anti-CD3.

### In vivo transfer colitis model

IL-10^eGFP+^Foxp3^mRFP−^ cells from spleen or small intestine (Fig. 1) or IL-10^eGFP+^Foxp3^mRFP−^CD49b^+^LAG-3^+^ and IL-10^eGFP+^Foxp3^mRFP−^ not CD49b^+^LAG-3^+^ double positive cells from spleen (Fig. 4) of anti-CD3 treated mice (CD45.2 Foxp3^mRFP^ IL-10^eGFP^ double reporter mice) were injected i.p. (50.000 cells/mouse) into lymphopenic hosts (CD45.1 *Rag1*−/−). The weight and endoscopic colitis score were monitored.

### In vivo suppression colitis model

IL-10^eGFP+^ Foxp3^mRFP-^ CD49b^+^LAG-3^+^ cells and Foxp3^+^ cells of anti-CD3 treated Foxp3^mRFP^, IL-10^eGFP^ reporter mice (CD45.2) were injected i.p. (50.000 cells/mouse) into lymphopenic hosts (*Rag1*−/− CD45.1) together with IL-10^neg^ cells (50.000 cells/mouse) isolated from the same animals. The weight and endoscopic colitis score was assessed 5 weeks after transfer.

### Plasmodium berghei infection ANKA (PbA)

Pre-experimental mice were i.p. injected with PbA (2 × 10^6^ infected erythrocytes) and blood was harvested 6 days post infection. Infected erythrocytes were used to infect the experimental mice (1 × 10^5^ infected erythrocytes/mouse). Animals were killed for analysis on day 6 post infection. To assess the level of parasitemia, a blood smear was stained using WRIGHT stain.

### Endoscopic procedure

Colonoscopy was performed in a blinded fashion for colitis monitoring using the Coloview system (Karl Storz, Germany). Colitis scoring was based on granularity of mucosal surface, stool consistency, vascular pattern, translucency of the colon, and fibrin visible (0–3 points for each)^11,28,30^.

### In vitro suppression assay (mouse)

Responder T cells (CD4^+^ CD25^−^) were isolated from C57BL/6 wildtype mice using MACS beads (Miltenyi) and labeled with 5 µM violet dye. Responder cells were activated in the presence of irradiated APCs and 1.5 µg anti-CD3 (clone 2C11) either alone or combined with the indicated suppressor population in a ratio of 1:1 for 4 days. Proliferation of responder T cells was measured via flow cytometry.

### Lymphocyte isolation from human intestinal tissues

Tissue specimens were collected directly during the endoscopy, as is stated later on in the study approval section. After being cleaned with PBS, the tissue was digested in 5 ml full media containing collagenase IV (100 U, Sigma-Aldrich) for 20 min at 37 °C. The cells were further separated by a Percoll gradient (GE Healthcare).

### PBMC isolation from buffy coats

PBMCs were isolated from buffy coats of healthy donors using Biocoll separation solution (Biochrom). CD4^+^ T cells were further enriched using MACS CD4 beads (Miltenyi).

### In vitro suppression assay (human)

IL-10^pos^CD49b^+^LAG-3^+^ (CD25Low) and IL-10^pos^ not CD49b^+^LAG-3^+^ double positive (CD25Low) CD4 T cells were sorted using a FACSAria II. In parallel, responder CD4 T cells were sorted from unstimulated PBMCs from the same donor (CD25lowCD127high). Responder T cells were labeled with CFSE according to manual (2 µM, 8 min, 37 °C). Responder T cells and suppressor T cells were cultured in a 1:1 ratio in full media in the presence of Treg inspector beads (Miltenyi) at a ratio of 3 beads/cell for 4 days.

### Relative gene expression analysis

RNA from cells was isolated using TRIzol LS reagent (Life Technology) according to the manual. RNA was subjected to reverse transcription with SuperScript II (Invitrogen) with oligo(dT) primer. cDNA was semi quantified using commercially available primer/probe sets from Applied Biosystems. Samples were analyzed with the change in cycle threshold method. Results were normalized to hypoxanthine phosphoribosyltransferase (*Hprt*), quantified in parallel amplification reactions.

### Bulk-seq read processing

Single-end sequencing reads were trimmed using Trimmomatic followed by Alignment using Tophat and summarized with HTSeq count.

### 10XGenomics Drop-seq read processing

Pair-ended UMI-indexed Illumina reads were demultiplexed using cellranger mkfastq pipeline, trimmed using Trimmomatic followed by standard CellRanger pipeline in order to perform alignment to the respective genome (Mm10 or Hg19), read filtering and UMI counting for each sample. UMI count tables were imported into R environment (RStudio) for further data analysis.

### SMART-seq2 read processing

Pair-ended whole-transcript Illumina reads were demultiplexed using de-indexed and aligned to the Mm8 mouse genome through 2-pass alignment using STAR^73^. RPKM expression values were computed using uniquely aligned reads^74^ with MULTo correction^75^.

### Bulk RNA-seq data analysis

A standard RNAseq pipeline^76,77^ was used for data analysis of bulk-sorted cell populations (IL10^pos^CD49b^+^LAG-3^+^, IL10^pos^ not CD49b^+^LAG-3^+^ double positive, IL10^neg^ not CD49b^+^LAG-3^+^ double positive). Briefly, mitochondrial genes were excluded from the analysis as well as genes that fail to have over 5 counts in more than 4 samples. Data normalization, model fitting, and computation of fold changes were done using DESeq2^77^. Genes with fold change above 1.5 and FDR below 1% were considered differentially expressed between each pairwise comparison.

### Single cell data analyses

A similar analysis strategy^78^ was applied to both SMARTseq-2 and 10XGenomics datasets and is described briefly as follows. Cells were filtered out based on the library size, number of genes, and mitochondrial gene proportion using 3 median absolute deviations below the median log-library size for each parameter. Cell classification was performed using Cyclone^79^ in order to filter out dividing G2/M cells. Genes with mean row counts below 1 (0.05 for 10 × ) were considered to be of low-abundance and were removed. Data normalization was performed using pool counts method^80^ and using ERCC spike-in correction when available. Normalized log-expression values were used for identification of HVGs with biological variance greater than 0.5 (0.05 for 10 × ) and false discovery rate of 5%. Normalized log-expression values of HVGs were subsequently processed for non-linear dimensionality reduction using Rtsne (http://CRAN.R-project.org/package=Rtsne) and further clustering using DBSCAN (https://CRAN.R-project.org/package=dbscan/) on tSNE space. Differential gene expression among clusters was done using SCRAN^78^ and Gene Ontology enrichment analysis was performed using EnrichR. Annotation of cytokines, transcription factors, and receptors used are a consensus of several gene annotation databases. Plots were generated using R base graphics, heatmap, vioplot and RColorBrewer (https://CRAN.R-project.org/package=pheatmap; https://CRAN.R-project.org/package=vioplot; http://CRAN.R-project.org/package=RColorBrewer).

For correlation between mouse single cell clusters from both spleen and small intestine, gene averages per clusters were used. Non-parametric Spearman correlation was applied in all non-zero values between each clusters compared, individually. For correlations using bulk RNA-seq, T_R_1 cell (IL10^pos^CD49b/LAG-3) gene-wise counts were averaged and correlated iteratively to each cell using only the HVGs, respective to the single-cell dataset being used.

For comparing gene heterogeneity between spleen and small intestine single cell datasets, we opted to compute the difference in the number of HVGs in each dataset by bootstrap. For this purpose, 100 cells were randomly selected and used to compute the number of HVGs (as indicated above) for each dataset. This process was repeated 100 times with another 100 randomly picked cells, with replacement at each time, and the number of HVGs was recorded. Results obtained this way presented a normal distribution and were compared using two-tailed Student’s *t*-test. *P* values below 0.05 were considered significant.

### Statistics

For non-transcriptomic data, two-tailed Mann–Whitney *U* test, paired *t*-test, Wilcoxon test, or one-way *ANOVA* (with Tukey post-hoc test) test were used to calculate significance, where applicable. *P* values below 5% were considered significant. Statistical calculation was performed using Prism program 5.0 (GraphPad Software).

### Study approval

Experiments involving animals were carried out in accordance with the Institutional Review Board “Behörde für Soziales, Familie, Gesundheit und Verbraucherschutz” (Hamburg, Germany). Experiments were carried out in accordance to all relevant ethical regulations.

Human studies were approved by the local ethical committee (Ethik-Kommission der Ärztekammer Hamburg). Consent was obtained from all human participants and experiments were carried out in accordance with all relevant ethical regulations.

## Electronic supplementary material

Supplementary Information

Transparent Peer Review File

## Data Availability

All RNA-seq data generated is available at Gene Expression Omnibus (GEO) under the accession number GSE121393. All other relevant data are available from the authors.
