# Supplementary material for: Molecular and functional heterogeneity of IL-10-producing CD4+ T cells
Source: Nat Commun. 2018 Dec 21;9:5457. doi: 10.1038/s41467-018-07581-4 (PMC6303294; doi:10.1038/s41467-018-07581-4)
Supplement: Supplementary file 1 — Supplementary Information [file 41467_2018_7581_MOESM1_ESM.pdf]

Molecular and functional heterogeneity of IL-10-producing $CD4^+$ T cells

Brockmann et al.

Supplementary Information

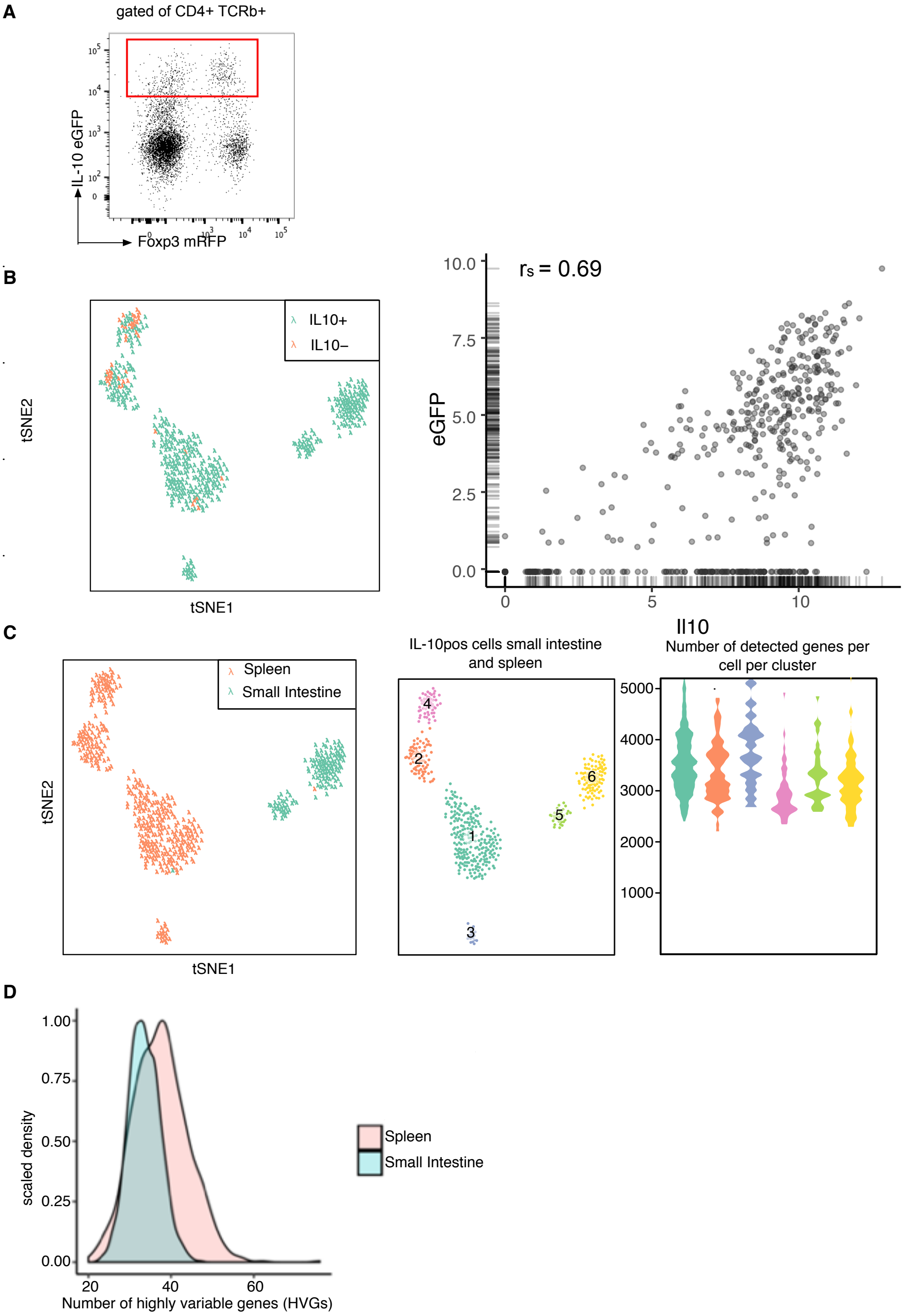

Supplementary Figure 1. IL-10-producing Foxp3^{Neg} CD4^{+} T cells are transcriptionally heterogeneous. A) Representative dot plot of IL-10^{eGFP} and Foxp3^{mRFP} expression in CD4^{+} T cells. Red gate indicates which cells were sorted for single cell sequencing analysis. **B)** Expression of *Il10* and correlation between *Il10* and *Gfp* expression. **C)** *t*-SNE analysis of single cell RNA sequencing (plate-based) of IL-10pos cells (including Foxp3^{+} Treg) isolated from small intestine or spleen of aCD3-treated IL-10^{eGFP} x Foxp3^{mRFP} double reporter mice. **D)** Bootstrap analysis of HVGs in single cell RNA sequencing data of IL-10pos cells from small intestine and spleen.

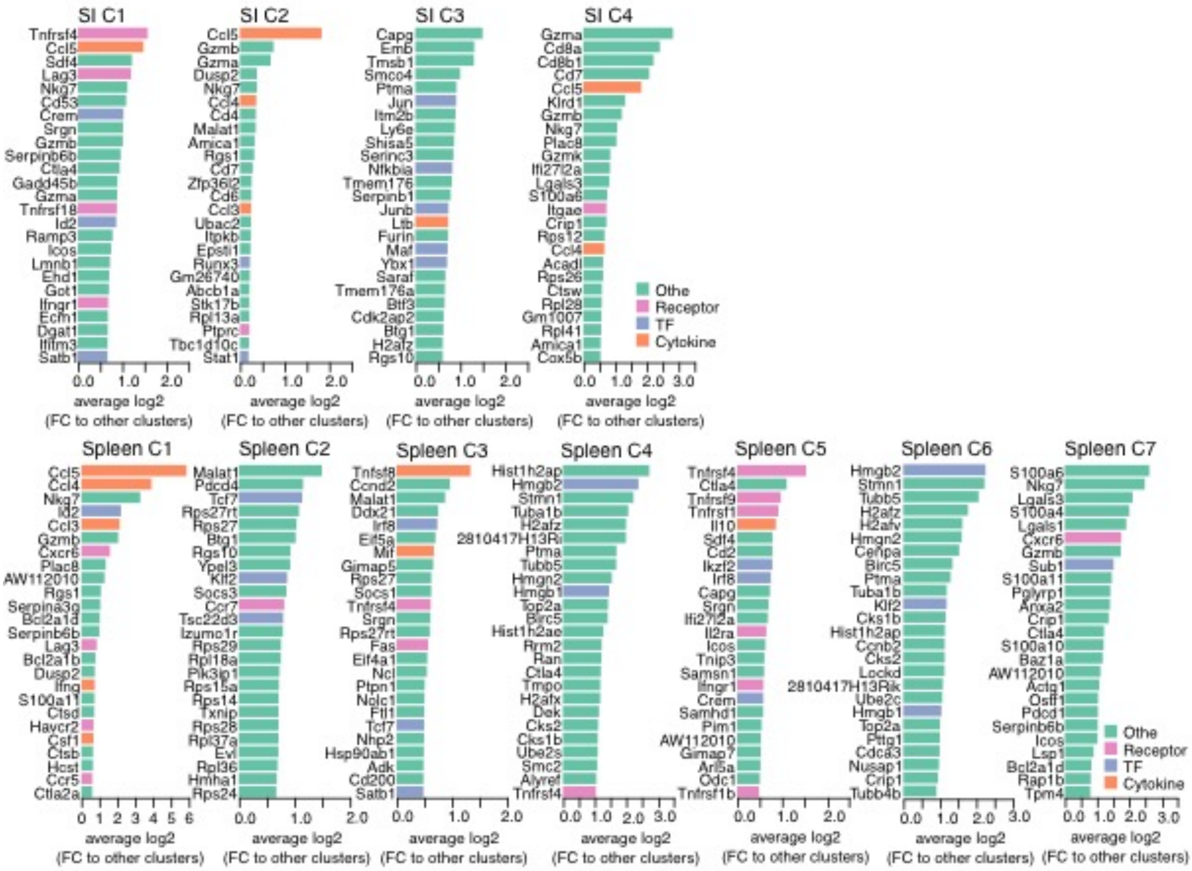

Supplementary Figure 2. Fold change analysis of 10X single cell RNA sequencing of IL-10-producing CD4$^+$ T from spleen and small intestine. Fold change (FC) of each cluster compared to other clusters. 20 most enriched genes for each cluster are shown.

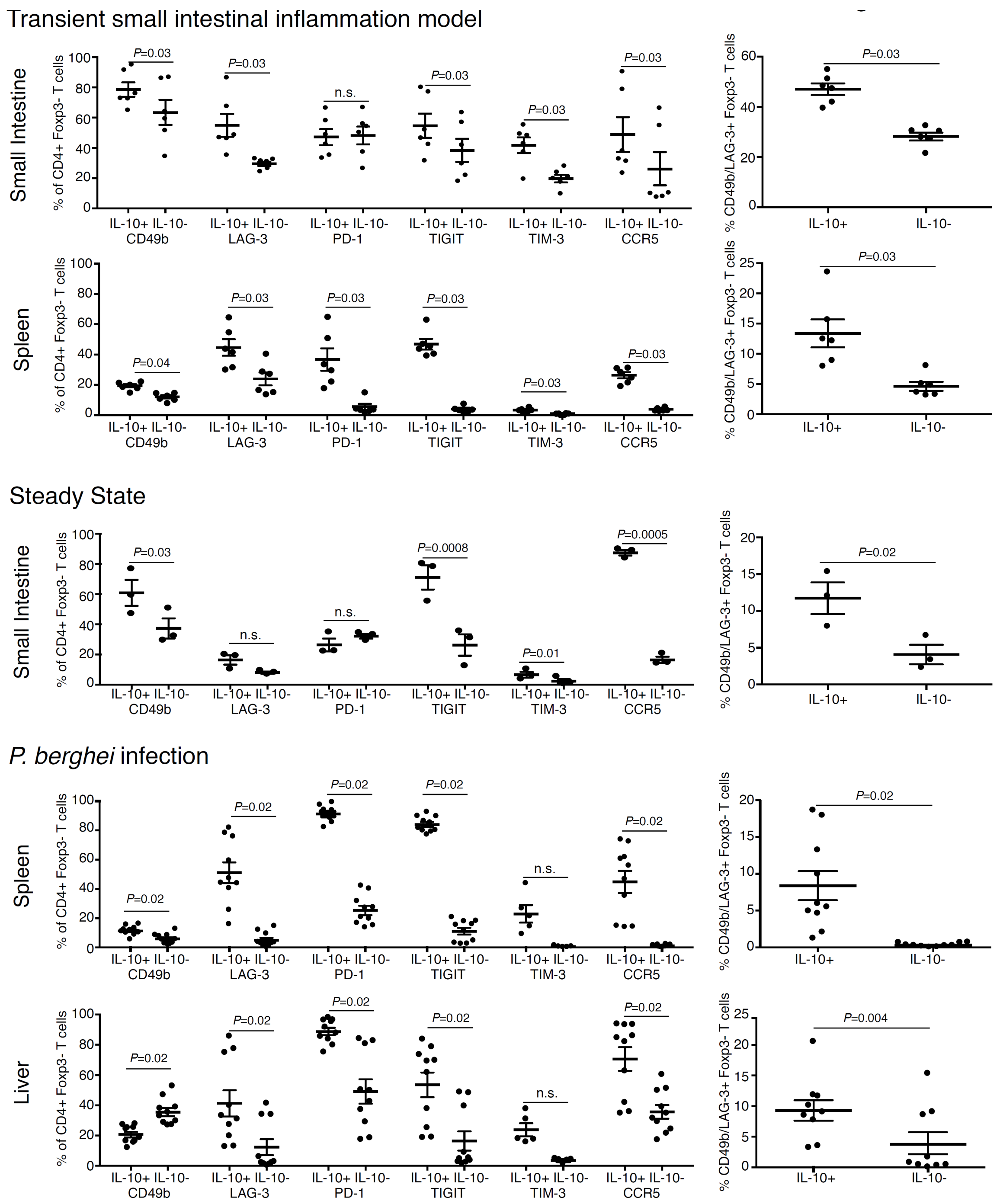

Supplementary Figure 3. Co-inhibitory receptors and T_R1-associated markers are differentially expressed on IL-10-producing $CD4^+$ T cells and IL-10 negative T cells. Analysis of cells from small intestine and spleen of aCD3-treated IL-10eGFP x Foxp3mRFP double reporter mice (n=6). Data are cumulative of two independent experiments. Analysis of untreated IL-10^{eGFP} x $Foxp3^{mRFP}$ double reporter mice (n=3). Data are representative of three independent experiments. Analysis of *P. berghei* infected IL-10^{eGFP} x $Foxp3^{mRFP}$ double reporter mice (n=10). Data are cumulative of three independent experiments. A paired t-test was used to calculate significance.

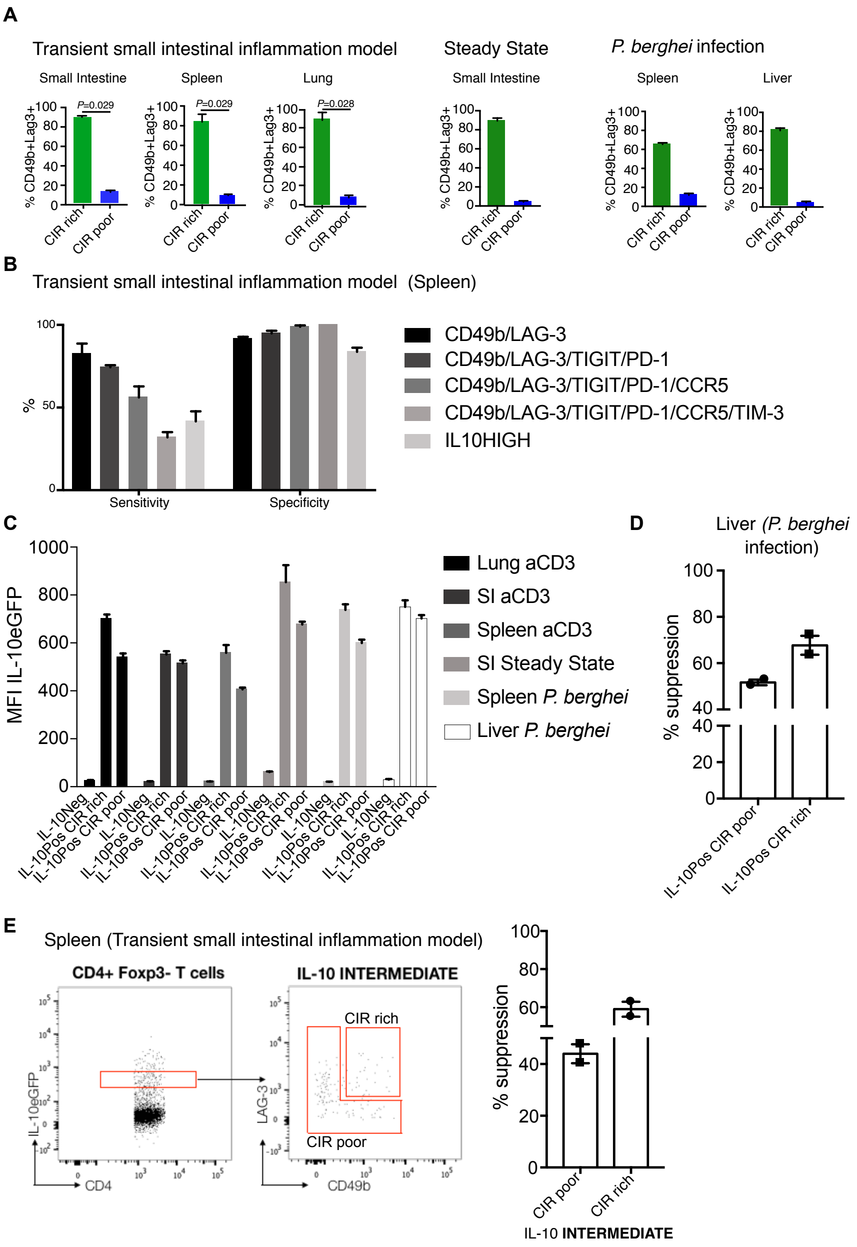

Supplementary Figure 4. CD49b/LAG-3 expression is strongly enriched within the IL-10-producing Foxp3^{Neg} CIR rich subpopulation and their superior suppressive capacity is independent of IL-10 expression A) Analysis of CD49b/LAG-3 co-expression in CIR rich and CIR poor subpopulations in indicated organs and disease models. **B)** Sensitivity and specificity was calculated for indicated marker combinations in IL-10-producing Foxp3^{Neg} CD4^{+} T cells derived from spleen of aCD3-treated IL-10eGFP x Foxp3mRFP double reporter mice. **C)** MFI of IL-10^{eGFP} assessed in IL-10-producing Foxp3^{Neg} CIR rich and CIR poor cells based on the analysis displayed in Figure 3A-C. Lymphocytes were isolated from spleen, lung and small intestine of aCD3-treated IL-10^{eGFP} x Foxp3^{mRFP} reporter mice. Additionally, cells were isolated from small intestine under steady state conditions and from spleen and liver of *P. berghei* infected mice. **D)** *In vitro* suppression of IL-10-producing Foxp3^{Neg} CIR rich and CIR poor CD4^{+} T cells isolated from liver of *P. berghei* infected IL-10^{eGFP} x Foxp3^{mRFP} reporter mice. Results are cumulative of two independent experiments. **E)** Representative gating strategy is shown (left). *In vitro* suppression of IL-10$^{\mathbf{INTERMEDIATE}}$ Foxp3^{Neg} CIR rich and CIR poor cells isolated of spleen of aCD3-treated IL-10^{eGFP} x Foxp3^{mRFP} reporter mice. Data are cumulative of two independent experiments.

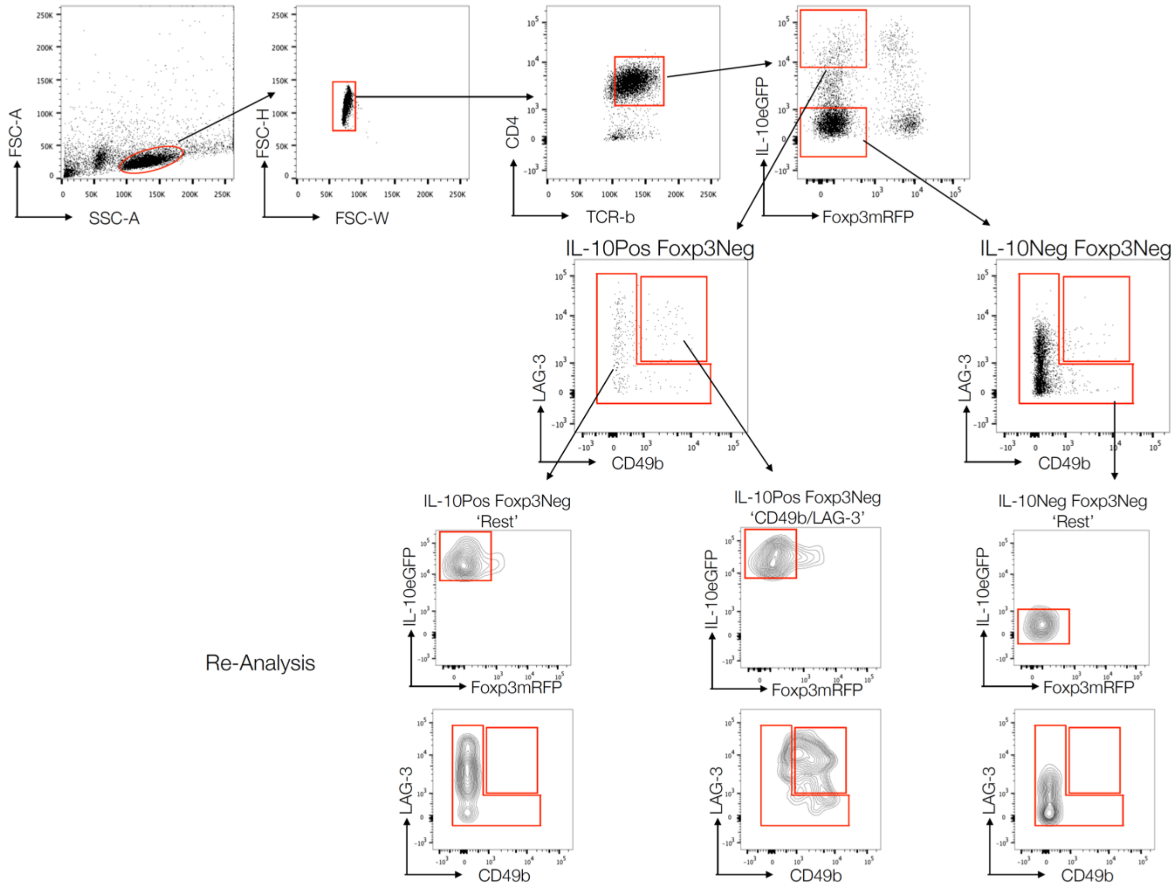

Supplementary Figure 5. Gating strategy used for analysis and sorting of IL-10-producing Foxp3^{Neg} CIR rich and CIR poor CD4^{+} T cells. Representative gating strategy of indicated populations is depicted in red as used in Figure 1D-F (IL-10pos Foxp3Neg cells), Figure 3 (IL-10pos, Foxp3Neg cells), Figure 4 (IL10pos CIR rich and CIR poor, IL10Neg CIR poor) and Figure 5A-C (IL10pos CIR rich and poor, IL-10Neg CIR poor). The representative plots show re-analysis of sorted populations.

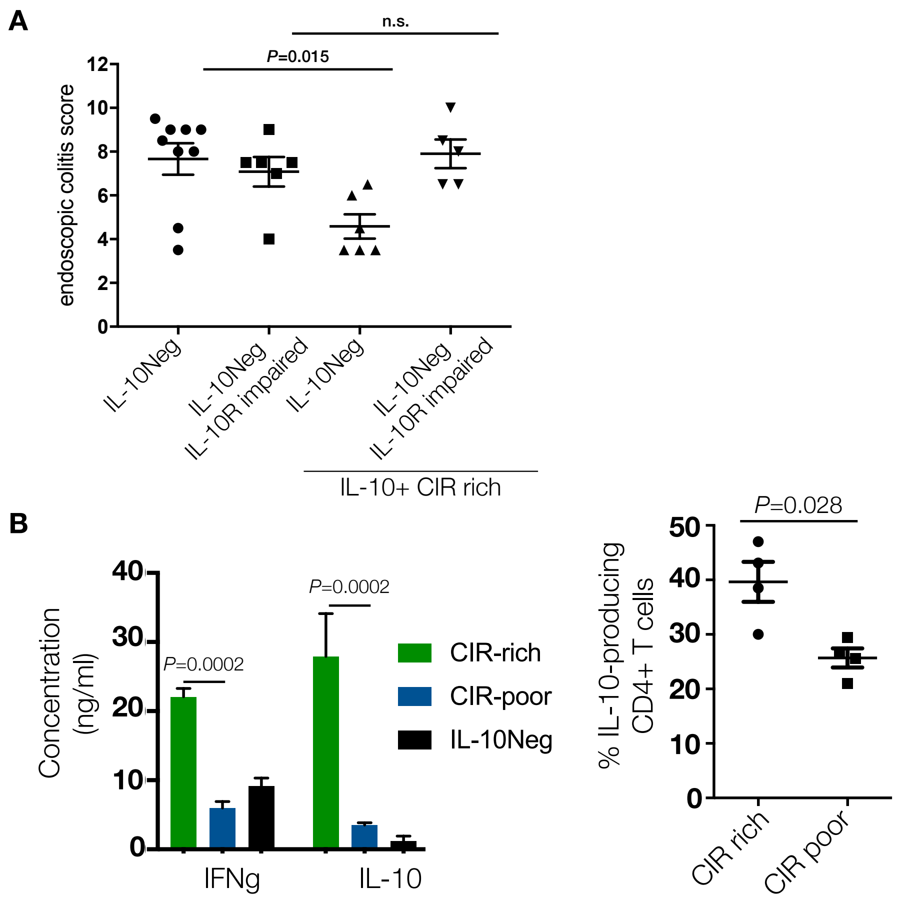

Supplementary Figure 6. Suppressive capacity of CIR rich cells is dependent on IL-10 during colitis *in vivo*. A) CIR rich IL-10-producing Foxp3^{Neg} CD4^{+} T cells, IL-10^{Neg} CD4^{+} T cells and IL-10R impaired IL-10^{Neg} CD4^{+} T cells were isolated from spleen of aCD3-treated IL-10^{eGFP} x Foxp3^{mRFP} reporter mice or IL-10^{eGFP} x Foxp3^{mRFP} IL-10R DN mice (IL-10R impaired). IL-10-producing CIR rich were co-transferred with IL-10^{Neg} CD4^{+} T cells or IL-10R impaired IL-10^{Neg} CD4^{+} T cells and colitis development was assessed by endoscopic colitis score 3 weeks upon transfer (IL-10Neg n=9; IL-10Neg IL-10R impaired n=6; IL-10Neg+CIR-rich n=6; IL-10Neg IL-10R impaired + CIR-rich n=5; lines indicate mean +/- SEM). Results are cumulative of three independent experiments. One-way ANOVA (post-test Tukey) was used to calculate significance. **B)** IL-10-producing Foxp3^{Neg} CIR rich and CIR poor cells were isolated from spleen of aCD3-treated IL-10^{eGFP} x Foxp3^{mRFP} reporter mice. Cells were re-stimulated with plate-bound aCD3 antibody and soluble CD28 antibody for 72 hours. IL-10^{eGFP} expression was assessed as well as cytokine concentration in the cell supernatant. Data are cumulative of four independent experiments. Mann Whitney test was used to calculate significance.

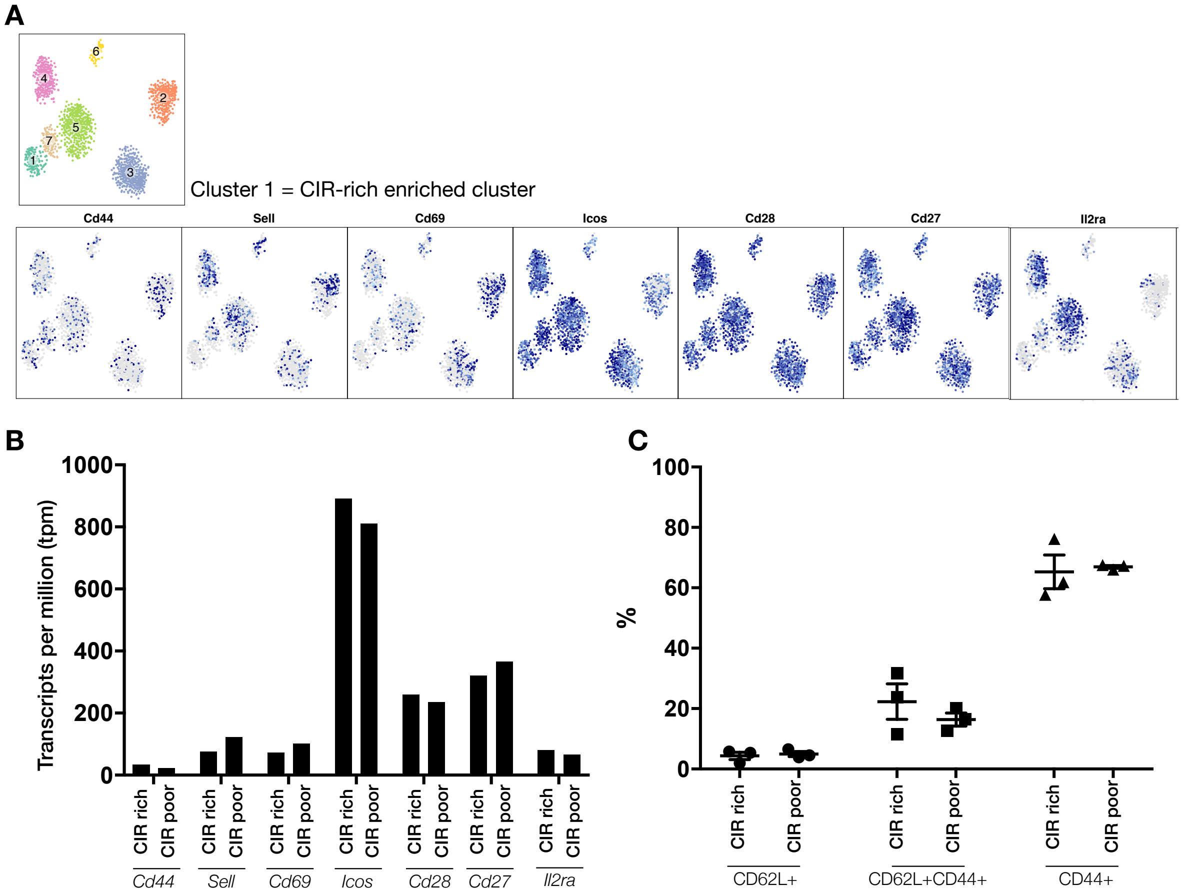

Supplementary Figure 7. Analysis of activation markers in single cell sequencing and FACS data. A) *t*-SNE analysis of single cell RNA sequencing of IL-10-producing cells (including $Foxp3^{+}$ cells) isolated from spleen of aCD3-treated $IL\text{-}10^{eGFP}$ x $Foxp3^{mRFP}$ reporter mice. Expression of indicated genes within cells of *t*-SNE analysis. Cluster 1 is enriched for CIR rich cells (see Figure 5). **B**) Transcripts per million (tpm) of indicated activation markers in transcriptome analysis of FACS-sorted CIR rich and CIR poor cells isolated from spleen from aCD3-treated $IL\text{-}10^{eGFP}$ x $Foxp3^{mRFP}$ reporter mice. **C**) Protein expression of CD62L and CD44 was analysis by flow cytometry in IL-10-producing $Foxp3^{Neg}$ CIR rich and CIR poor cells isolated from spleen of aCD3-treated $IL\text{-}10^{eGFP}$ x $Foxp3^{mRFP}$ reporter mice. Data are cumulative of two independent experiments.

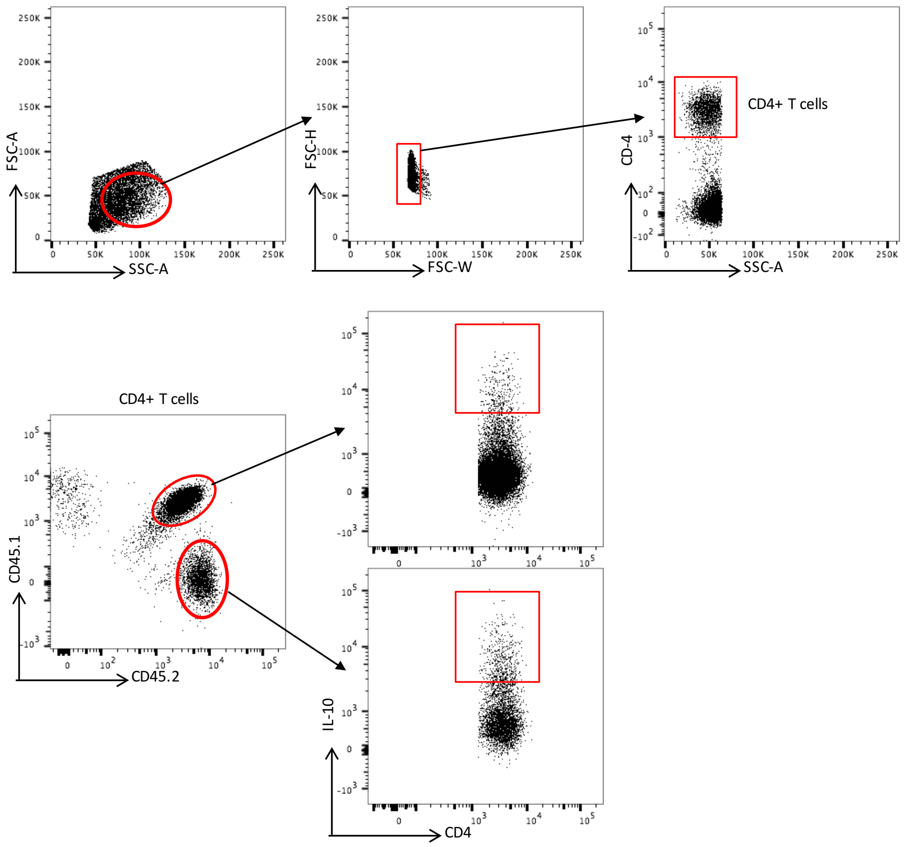

Supplementary Figure 8. Gating strategy used for analysis of LXRaKO and WT CD4^{+} T cells. Representative gating strategy of indicated populations is depicted in red as used in Figure 5D.

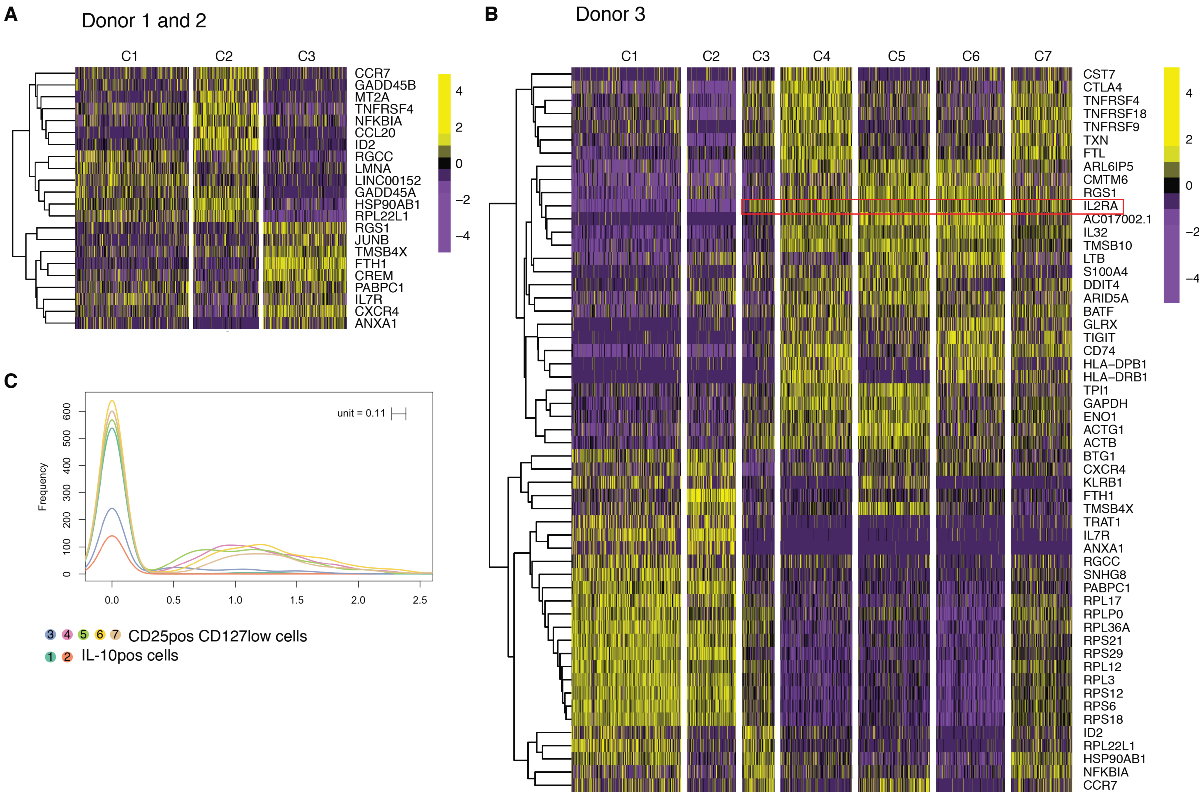

Supplementary Figure 9. Expression analysis of 10X single cell RNA sequencing of human IL-10-producing CD4^{+} T from PBMCs. A) Expression of highly variable genes in every cell in each cluster from donor 1 and 2. **B)** Expression of highly variable genes in every cell in each cluster from donor 3. **C)** Expression of *FOXP3* in Cluster 1-7 from donor 3.

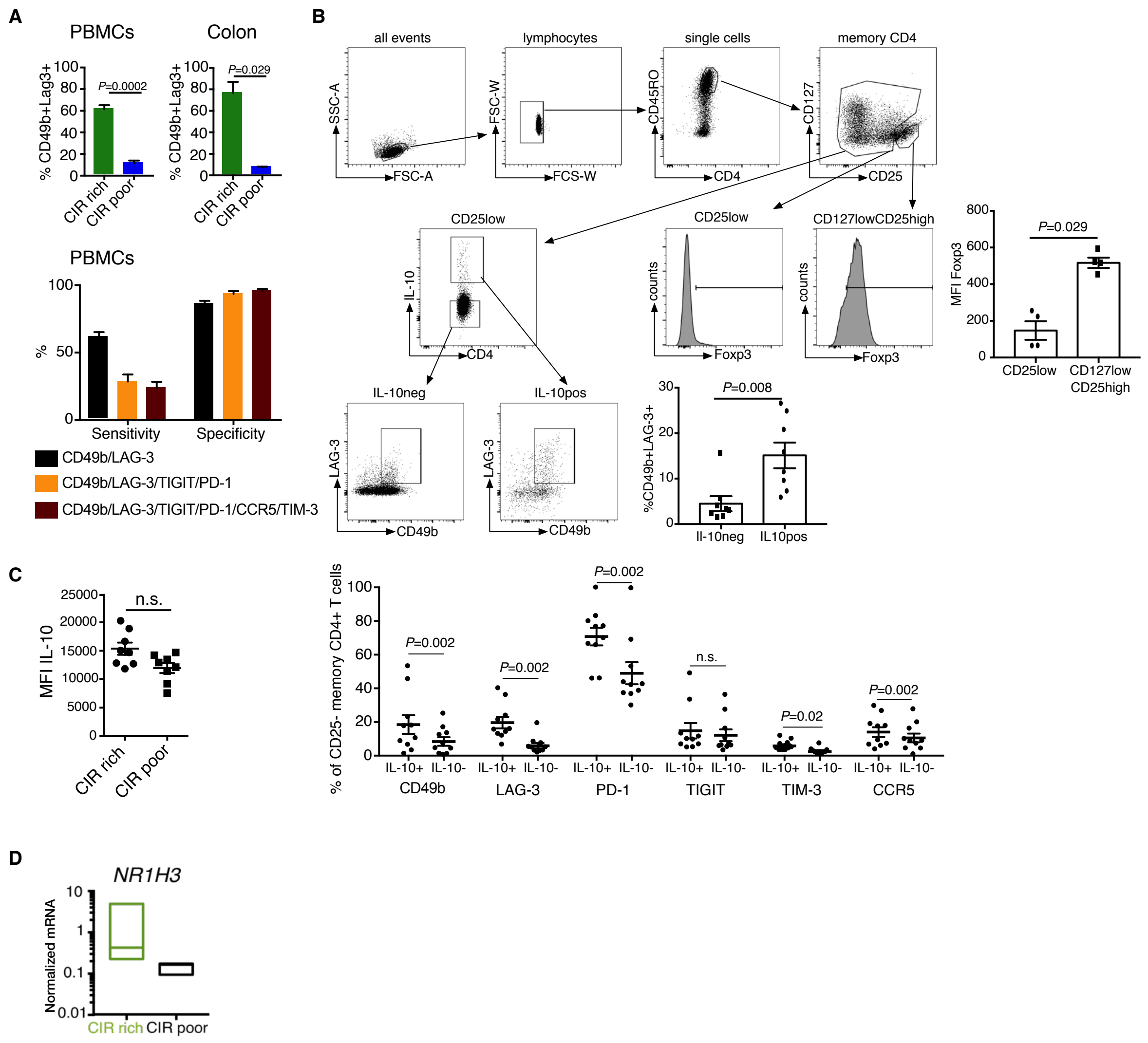

Supplementary Figure 10. CD49b/LAG-3 expression is strongly enriched within the IL-10-producing CD25^{Low} CIR rich subpopulation and FOXP3^{+} Treg cells are not contaminating CD25^{Low} memory CD4^{+} T cells. A) Analysis of CD49b/LAG-3 co-expression in IL-10-producing CIR rich and CIR poor subpopulations in indicated organs. Sensitivity and specificity was calculated for indicated marker combinations in IL-10-producing CD25^{Neg} CD4^{+} T cells derived from SEB-stimulated PBMCs of healthy donors. **B)** Gating strategy of IL-10-producing CD25^{Low} CD4^{+} T cells, CD49b/LAG-3 co-expression and expression of FOXP3 in CD4^{+} T cells isolated from PBMCs of healthy donors, re-stimulated overnight with SEB. Expression of indicated markers in IL-10-producing and IL-10 negative CD4+ T cells isolated from PBMCs of healthy donors, re-stimulated overnight with SEB. **C)** PBMCs were isolated from healthy donors (n=8) and stimulated overnight with SEB. IL-10-producing CD25^{Low} CIR rich and CIR poor cells were identified using *vi*SNE analysis as displayed in Figure 6B. IL-10 protein levels were assessed within those populations. Data are cumulative of 4 different experiments. **D)** PBMCs were isolated from healthy donors and re-stimulated overnight with 1 µg/ml SEB. CD4^{+} T cells were enriched using CD4 MACS-beads before sorting. IL-10-producing CD25^{Low} CIR rich and CIR poor cells were isolated using FACS. mRNA expression of *NR1H3* normalized to *HPRT* from three independent experiments is shown.

Patient information

	Controls (n=18)	UC-remission (n=2)	UC-active (n=22)	CD-remission (n=5)	CD-active (n=9)
Sex (F/M)	9/9	2/0	9/13	2/3	5/4
Age (years)*	54.5 +/- 18.1	39 +/- 25.5	42.8 +/- 15.03	43.4 +/- 12.7	31.4 +/- 12.8
Involvement UC					
left-sided			5		
Distal			11		
Pancolitis			3		
Involvement CD					
SI					3
Colon					5
SI + colon					1

* mean +/- SD

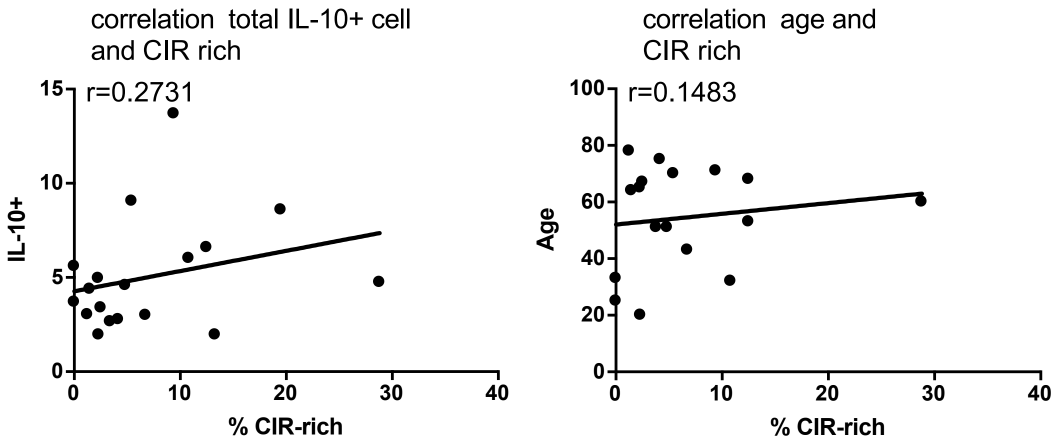

Supplementary Figure 11. Patient information and correlation between CIR rich cells and IL-10 expression and CIR rich cells and age in healthy controls.
