# Supplementary material for: Molecular and functional heterogeneity of IL-10-producing CD4+ T cells
Source: Nat Commun. 2018 Dec 21;9:5457. doi: 10.1038/s41467-018-07581-4 (PMC6303294; doi:10.1038/s41467-018-07581-4)
Supplement: Supplementary file 2 — Transparent Peer Review File [file 41467_2018_7581_MOESM2_ESM.pdf]

Editorial Note: This manuscript has been previously reviewed at another journal that is not operating a transparent peer review scheme. This document only contains reviewer comments and rebuttal letters for versions considered at Nature Communications.

REVIEWERS' COMMENTS:

Reviewer #1 (Remarks to the Author):

The authors well responded to the reviewer's comments, and the manuscript is now greatly improved. The reviewer has no additoional comments.

Reviewer #2 (Remarks to the Author):

The major concerns have largely been addressed.

REVIEWERS' COMMENTS:

Reviewer #1 (Remarks to the Author):

The authors well responded to the reviewer's comments, and the manuscript is now greatly improved. The reviewer has no additoional comments.

We thank Reviewer #1

Reviewer #2 (Remarks to the Author):

The major concerns have largely been addressed.

We thank Reviewer #2
